# Advancing breast cancer diagnosis: token vision transformers for faster and accurate classification of histopathology images

**DOI:** 10.1186/s42492-024-00181-8

**Published:** 2025-01-08

**Authors:** Mouhamed Laid Abimouloud, Khaled Bensid, Mohamed Elleuch, Mohamed Ben Ammar, Monji Kherallah

**Affiliations:** 1 National Engineering School of Sfax, University of Sfax, Sfax, Tunisia; 2 Laboratory of Electrical Engineering (LAGE), University of KASDI Merbah Ouargla, 30000 Ouargla, Algeria; 3 National School of Computer Science (ENSI), University of Manouba, Manouba, Tunisia; 4 Department of Information Systems, Faculty of Computing and Information Technology, Northern Border University, Rafha, Saudi Arabia; 5 Faculty of Sciences, Sfax, Tunisia; 6 Advanced Technologies for Environment and Smart Cities (ATES Unit), Sfax University, Sfax, Tunisia

**Keywords:** Breast cancer, Convolutional vision transformer, Histopathological images, Multi classification, Brekhis

## Abstract

The vision transformer (ViT) architecture, with its attention mechanism based on multi-head attention layers, has been widely adopted in various computer-aided diagnosis tasks due to its effectiveness in processing medical image information. ViTs are notably recognized for their complex architecture, which requires high-performance GPUs or CPUs for efficient model training and deployment in real-world medical diagnostic devices. This renders them more intricate than convolutional neural networks (CNNs). This difficulty is also challenging in the context of histopathology image analysis, where the images are both limited and complex. In response to these challenges, this study proposes a TokenMixer hybrid-architecture that combines the strengths of CNNs and ViTs. This hybrid architecture aims to enhance feature extraction and classification accuracy with shorter training time and fewer parameters by minimizing the number of input patches employed during training, while incorporating tokenization of input patches using convolutional layers and encoder transformer layers to process patches across all network layers for fast and accurate breast cancer tumor subtype classification. The TokenMixer mechanism is inspired by the ConvMixer and TokenLearner models. First, the ConvMixer model dynamically generates spatial attention maps using convolutional layers, enabling the extraction of patches from input images to minimize the number of input patches used in training. Second, the TokenLearner model extracts relevant regions from the selected input patches, tokenizes them to improve feature extraction, and trains all tokenized patches in an encoder transformer network. We evaluated the TokenMixer model on the BreakHis public dataset, comparing it with ViT-based and other state-of-the-art methods. Our approach achieved impressive results for both binary and multi-classification of breast cancer subtypes across various magnification levels (40×, 100×, 200×, 400×). The model demonstrated accuracies of 97.02% for binary classification and 93.29% for multi-classification, with decision times of 391.71 and 1173.56 s, respectively. These results highlight the potential of our hybrid deep ViT-CNN architecture for advancing tumor classification in histopathological images. The source code is accessible: https://github.com/abimouloud/TokenMixer.

## Introduction

Breast cancer (BC) is one of the most severe and prevalent diseases and the second most common cancer worldwide [1]. In 2020, BC affected an estimated 2.3 million women globally, resulting in 685,000 deaths. Additionally, 7.8 million women were diagnosed with BC over the five-year period leading up to 2020 [2]. In clinical practice, dependence on expert-based diagnoses poses a significant challenge in the medical field, particularly in the manual and visual assessment of medical conditions.

The subjective nature of expert pathologist opinions can lead to variations in diagnoses, affecting the overall accuracy and reliability of the diagnostic process [3]. In addition, late detection of tumors may result in serious cases of BC being misclassified as benign, leading to a failure to detect cancer at an early stage. This delay can allow malignant cancers to develop and spread throughout the body. Various imaging modalities have been used in medical image analysis, including X-rays [4], ultrasound [5], and magnetic resonance imaging (MRI) [6]. Recently, histopathology images (microscopic images) have become widely used for the diagnosis and analysis of cancers [7], providing insights into the nature of tissue areas [8, 9]. These images offer substantial information about malignant tissue, aiding in the extraction of features from microscopic examinations and providing comprehensive information [10].

In recent years, convolutional neural network (CNN) models have been extensively used to analyze medical images [11]. The integration of deep-learning techniques in the medical field assists pathologists in developing treatment plans for patients [12]. While CNNs excel at extracting local features from medical images, their ability to capture intricate relationships within tumors for classifying BC subtypes is limited. A significant challenge for CNN models is effectively localizing tumor regions in complex images [13]. Additionally, CNNs struggle with rotation and scale invariance, which calls for methods such as data augmentation, data preprocessing, and filters to extract image features. These problems become more pronounced when CNNs process medical breast images without focusing on specific regions of interest, leading to disadvantages in localization and accuracy [14]. Vision transformers (ViTs) and CNNs face challenges in achieving optimal performance to obtain robust neural networks that can extract features from images, which are constrained by the limited availability of data and computing resources [15]. ViTs which are known for their attention mechanism, excel in capturing long-range dependencies between image patch features [16]. In certain tasks, ViTs may overlook important local details that are essential for medical diagnoses, such as subtle tumor margins or distinct tissue textures. Moreover, the computational cost of training and running ViTs is higher because they require significant CPU and GPU resources to apply the attention function to the multi-head attention layer, unlike the convolution layers in CNNs [17]. In recent literature, research has shown significant interest in combining CNN models with ViT to address the challenges of minimizing computational resources and processing time required for training medical datasets, which is promising for enhancing the efficiency of tasks relating to BC histopathological image classification [18].

Thus, the main objective of this study was to develop TokenMixer, a new hybrid system that integrates an attention mechanism and convolution layers to reduce the decision time and resources required efficiently, without compromising the performance accuracy. The proposed architecture, based on TokenLearner, adopts an innovative approach to tokenize input patches through an adaptive tokenization mechanism. Subsequently, the ConvMixer layers dissect the selected patches into precise local features using convolution layers. This selection of tokens effectively reduces the total number of tokens for the patch images input to the ViT encoder. This fusion enables TokenMixer not only to identify tumor characteristics but also to comprehend their interplay. The result is more accurate and faster classification, leveraging the strengths of both tokenization strategies and convolution analysis with the attention mechanism. This study explored the feasibility of combining the strengths of CNNs and ViTs through tokenization mechanisms. The aim is to develop hybrid networks that are optimized for enhanced feature extraction and efficient decision-making while minimizing the computational resources of GPUs and decision time in histopathology image classification tasks. This study makes four significant contributions to the literature:

- The integration of CNNs and ViTs is explored to construct hybrid networks. This integration, aiming to achieve synergy between the two architectures. This approach has the potential to enhance the overall performance and effectiveness of medical data analysis.
- The proposed architecture combines CNNs and ViTs with the aim to reduce hardware device resources and training time for the classification of BC using histopathological images, facilitating timely decision-making.
- The model’s performance is extensively examined and compared with that of the ViT base model and other approaches in recent literature.
- The TokenMixer model approach extends beyond theoretical development, striving to offer practical advancements in the medical field.

The remainder of this paper is organized as follows: literature studies are provided in Literature survey subsection, and suggested methods are presented in Methods section. Results section presents the results, Discussion section provides a general discussion, and lastly, Conclusions section concludes with conclusions and future work.

### Literature survey

The applications of ViTs with attention mechanisms have gained considerable traction in the field of medical computer vision. Many architectures have been proposed and implemented for diverse tasks, including classification [19], segmentation [20], and cancer detection [21]. In 2021, Matsoukas et al. [22] posed an important question: Is it time to replace CNNs with Transformers for medical images? To address this question, the authors confirmed that transformers could effectively substitute CNNs for medical image tasks with minimal effort. ViTs have demonstrated the ability to match or even surpass the performance of CNNs, particularly in the context of small medical datasets. As to the same question, Henry et al. [15] posed a question regarding whether the impact of transformers on computer vision is translated to medical imaging in 2022. Their inquiry prompted a comprehensive exploration resulting in a detailed review of the applications of ViTs in medical imaging. The researchers performed a comprehensive comparative study to assess how ViTs compared to CNNs across a range of medical imaging techniques. In 2023, He et al. [23] aimed to raise awareness about the applications of ViTs in the medical image domain. Their focus was on emphasizing that the majority of existing transformer based methods can be seamlessly and straightforwardly applied to various medical imaging problems with minimal modifications. BC image classification has attracted considerable attention. Several studies have specifically focused on classifying breast tumors using histopathology images from the BreakHis dataset, aligning their objectives with the focus of the current study. A major challenge in using histopathology images with different magnification levels is the complex nature of the images and the need to resize them to a suitable size. Resizing images of different magnifications can lead to loss of image information. To address this issue, one solution technique proposed is to split the input images into non-overlapping sub-images known as patches or tokens.

However, using all of the patches for training can lead to increased computational costs and complexity. This problem led Ahmad et al. [24] to present an approach based on only discriminative patches being selected to train the network, which can reduce the computational burden. The authors adopted a patch selection technique based on a CNN and clustering. They classified histopathology images using whole-slide images and discriminative tokens. They utilized the EfficientNet architecture to extract features and a support vector machine (SVM) as output classifier.

Recently, Abimouloud et al. [25] aimed to reduce the complexity of multi-head attention layers in ViT models for BC tumor classification. They proposed hybrid models combining CNN and ViT layers, namely the compact convolutional transformer (CCT) and mobile vision transformer (MViT), for both binary and multi-tumor classification tasks. The authors demonstrated a significant method to integrate convolutional layers, which enhanced feature extraction from images, thereby reducing the reliance on multi-head attention layers. This approach maintained the performance of ViT models while decreasing complexity and training time, ultimately yielding accurate results. Sriwastawa and Arul Jothi [26] explored the effectiveness of various ViT models for classifying BC images into binary classification. They examined eight different ViT architectures, ranging from the original ViT [16] to convolutional vision transformer (CvT) [27], crossformer [28], crossViT [29], NesT [30], PiT [31], MaxViT [32], and separable vision transformer (SepViT) [33]. The authors used two medical image datasets: BreakHis and IDC. Their approach involved a two-step process. Initially, they trained each ViT model using the BreakHis dataset. Following this, they took these BreakHis-trained models and fine-tuned them using the IDC dataset.

Tummala et al. [34] proposed an ensemble model comprising a Swin transformer for BC classification using histopathological images. The Swin transformer is a hierarchical ViT that uses shifted windows. To enable efficient modeling, the self-attention is computed within local windows, which are arranged in a non-overlapping manner to partition the image evenly. This window-based self-attention has linear complexity and is scalable. However, the modeling power of window-based self-attention is limited owing to the lack of connections across windows. To address this issue, the authors proposed a shifted window partitioning approach that alternates between different partitioning configurations in consecutive Swin transformer blocks. This allows for cross-window connections while maintaining the efficient computation of non-overlapping windows. The shifted window scheme in Swin transformers offers increased efficiency by restricting the self-attention computation to local, non-overlapping windows, while also facilitating connections across windows. Overall, with this proposed method for BC classification, the Swin transformer network performed better than regular ViTs. Boumaraf et al. [35] proposed a transfer learning approach for automated classification of BC from histopathological images. They utilized a pretrained CNN on the ImageNet dataset, ResNet-18, as the backbone model for this task. The authors introduced a transfer learning method based on a block-wise fine-tuning strategy on ResNet-18 model to transfer the knowledge learned from natural ImageNet images to histopathological images. To accomplish this, the last two residual blocks of the fine-tuned ResNet-18 model were made trainable, thereby enabling them to capture the most representative task-specific features of the BreakHis dataset.

Joseph et al. [36] presented an approach for combined handcrafted feature extraction methods and deep neural networks for the multi-classification of BC using histopathological images from the BreakHis dataset. They employed handcrafted feature extraction techniques (Hu moment, Haralick textures, and color histograms) to extract features from images. These extracted features were then utilized to train deep neural network classifiers consisting of four dense layers and a Softmax output layer. Srikantamurthy et al. [37] introduced a hybrid model that combined a CNN and a long short-term memory recurrent neural network (LSTM RNN) for BC classification. The proposed hybrid CNN-LSTM model leverages a pretrained transfer learning model (either InceptionResNetV2 or ResNet50) until the final convolutional layer, which provides bottleneck features of a specific size. In parallel, the independent RNN module consists of two LSTM layers of a certain size. The outputs from the CNN and RNN modules are then merged using element-wise multiplication. This combined output is fed into a classification layer with eight nodes (corresponding to eight classes) and a Softmax activation function. The rationale behind this approach is that the convolutional layers with different filter sizes can capture images of various magnifications. Amin and Ahn [38] proposed FabNet lightweight CNN network for BC histopathological image classification. It uses an architecture to learn features from fine to coarse in multi-magnification histopathological images. The model consolidates hierarchical feature maps effectively. Its performance was demonstrated using a combination of NCT-CRC-HE-100 and BreakHis datasets. Hao et al. [39] proposed an approach that combines deep semantic features method with gray-level co-occurrence matrix (GLCM). They utilized a pretrained DenseNet201 as the base model and employed SVM for data classification. This was achieved by extracting deep semantic features from the convolutional layer features of the last dense block, which were then merged with the three-channel GLCM features.

Mahmud et al. [40] utilized transfer learning and deep feature extraction techniques, and leveraged AlexNet for additional fine-tuning. They modified the pretrained CNN models AlexNet and VGG-16 for feature extraction. Subsequently, the extracted features were fed into SVMs for classification tasks. Abunasser et al. [41] introduced a deep-learning model that incorporated several fine-tuned models such as Xception, InceptionV3, VGG-16, MobileNet, ResNet50, and BCCNN. The authors proposed an approach that combined these pretrained models to enhance the overall performance of their deep-learning solution. While previous research has made notable advancements in BC histopathological image classification (Table 1), the proposed CNN and ViT models often suffer from high complexity and a large number of parameters. Consequently, these models demand substantial hardware device resources and extended training times. This study addresses these limitations by introducing TokenMixer, a hybrid ViT-CNN model. TokenMixer capitalizes on the tokenization and patch extraction capabilities of CNN layers combined with multi-head attention layers of ViT, while maintaining a reduced parameter count and shorter training duration. Importantly, the proposed model trains from scratch without applying any preprocessing techniques. Through rigorous comparisons with recent similar studies, our goal is to introduce an innovative approach in the domain of BC histopathological images classification.

**Table 1:** Summary of related works studies

**Reference**	**Method**	**Data**	**Classification**
Ahmad et al. [24]	Patch selection approach based on EfficientNet and SVM classifier	BreakHis	BC
Abimouloud et al. [25]	ViT, CCT, MViT models	BreakHis	BC
Sriwastawa and Arul Jothi [26]	Fine-tuned ViT models	BreakHis IDC	BC
Tummala et al. [34]	Swin transformer models	BreakHis	BC
Boumaraf et al. [35]	Block-wise fine-tuning strategy based on a ResNet-18 model	BreakHis	BC
Joseph et al. [36]	Extract features based on Hu moment, Haralick textures, and color histogram	BreakHis	BC
Srikantamurthy et al. [37]	Feature extraction combines of LSTM RNN (InceptionResNetV2, ResNet50)	BreakHis	BC
Amin and Ahn [38]	FabNet model to learn fine-to-coarse structural and textural features	BreakHis NCT-CRC-HE-100	BC
Hao et al. [39]	Extract features based on DenseNet201 and SVM as classifiers	BreakHis	BC
Mahmud et al. [40]	Feature extraction based on AlexNet, VGG16, SVM classifiers	BreakHis	BC
Abunasser et al. [41]	Fine-tune deep learning models	BreakHis	BC
Our approach	Tokenization and patch extraction based on ViT-CNN	BreakHis	BC

## Methods

**Fig. 1:**
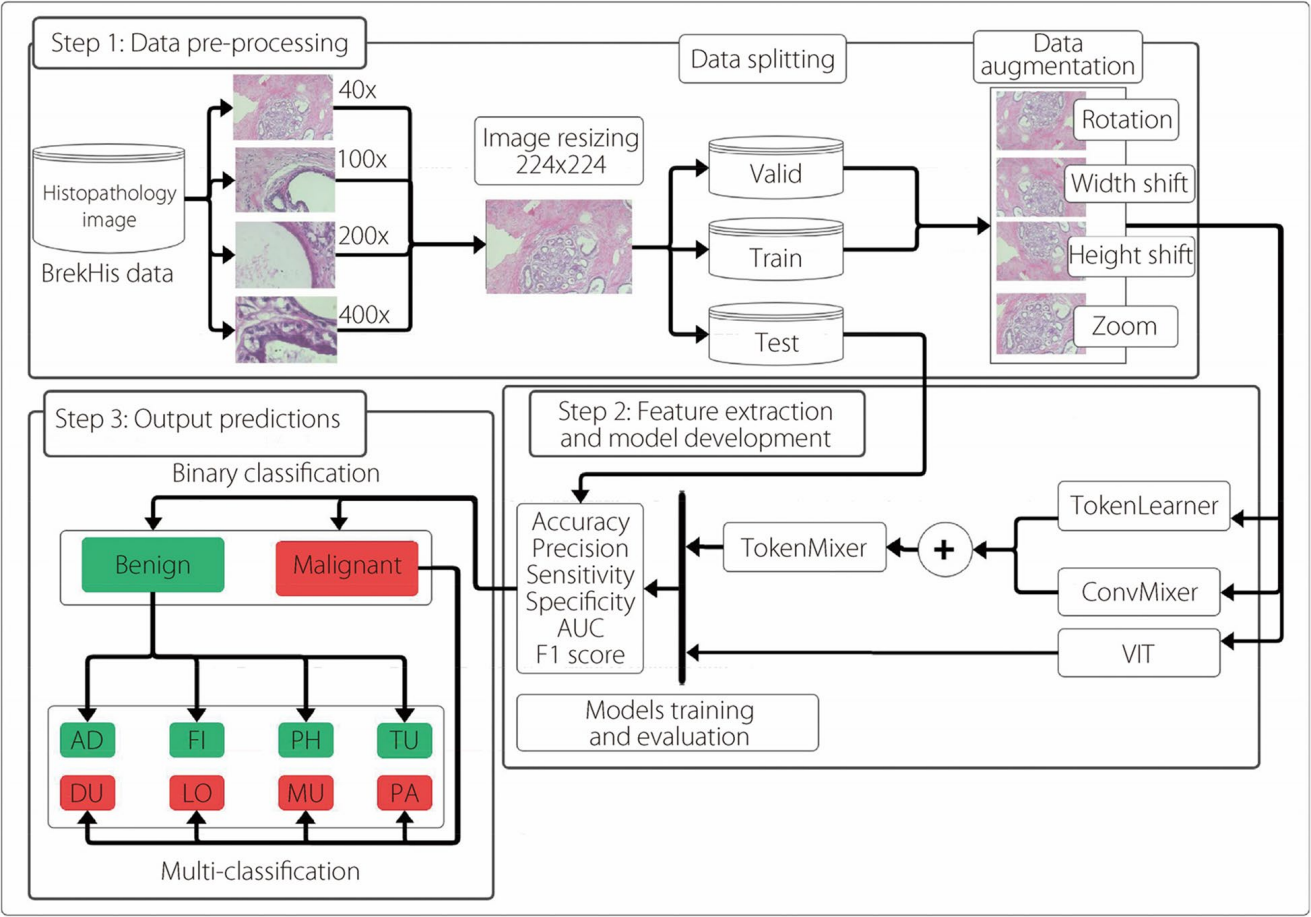
The structural steps of proposed method for BC histopathological images classification

This section outlines the development process of the proposed method, aimed at enhancing the accuracy of BC classification. Figure 1 shows the structural steps of our proposed method. The first step involves data preprocessing, in which histopathological images from the BreakHis dataset, captured at different magnification levels, are resized to a standardized size of 224 × 224 pixels. Subsequently, the data is split into three parts: training, validation, and test sets for each magnification sample. Additionally, we use data augmentation techniques such as rotation, width shift, height shift, and zoom. The second step involves feature extraction and model development using the preprocessed models: (1) TokenLearner: Responsible for learning image features and tokens; (2) ConvMixer: Applies convolutional layers to mixed tokens; (3) TokenMixer: Combines and mixes the learned tokens; and (4) ViT: A transformer based architecture used for comparison and validation of the proposed method.

The final step involves predicting the output, in which the TokenMixer model performs two classification tasks: (1) Binary classification: Distinguishing between ‘Benign’ and ‘Malignant’ images; (2) Multi-classification: The classification of images into eight tumors.

Throughout the training process, we trained all models from scratch, and we calculated various evaluation to evaluate and compare the performances of the different models on both the validation and test sets. The objective of our proposed method is to refine TokenMixer into an accurate and robust model for histopathological image classification, thereby facilitating BC diagnosis and treatment.

### Dataset

**Fig. 2:**
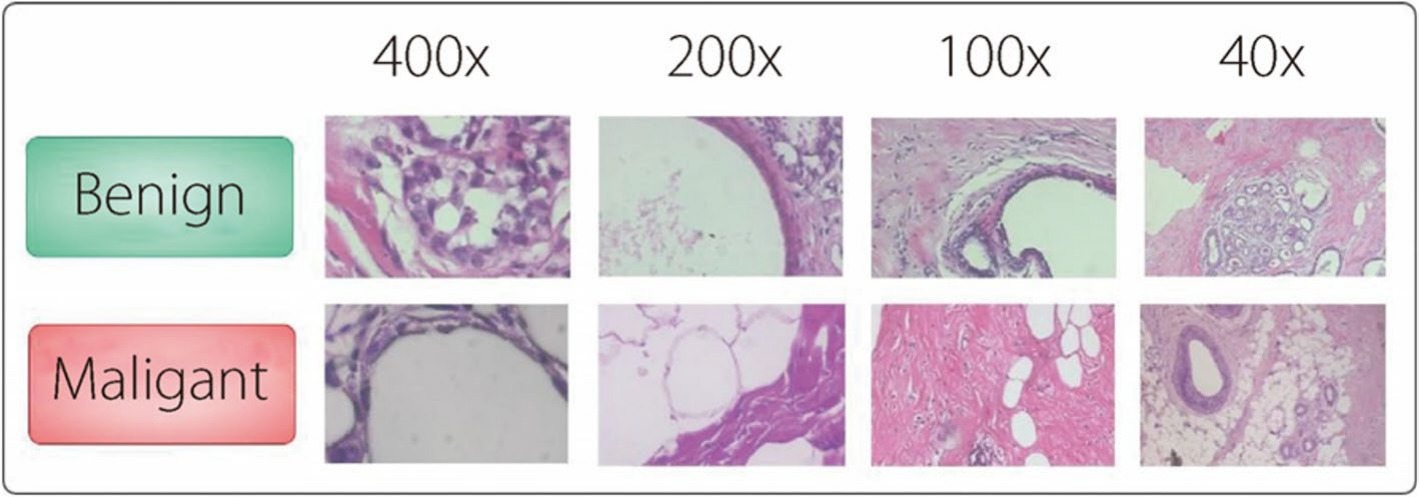
Images from the BreakHis dataset

This study utilizes the BreakHis dataset, which was presented by Spanhol et al. [42]. This publicly available dataset can be accessed from ref. [43]. Considering 7925 microscope images, the dataset includes 2496 benign and 5429 malignant breast tumors (Table 2). Four distinct histopathological subtypes were identified among the benign breast tumors: adenocarcinoma (AD), fibroadenoma (FI), phyllodes tumor (PH), and tubular adenoma (TU). The malignant breast tumors, which are categorized as BC, comprise four subtypes: ductal carcinoma (DU), lobular carcinoma (LO), mucinous carcinoma (MU), and papillary carcinoma (PA), as shown in Table 3. Additionally, Fig. 2 shows a sample image from the dataset. These images were captured at four different magnification levels: 40× , 100× , 200× , and 400× , in three-channel RGB format.

**Table 2:** The number of BreakHis data samples by each magnification for benign and malignant tumor classes

Magnification level	400×	200×	100×	40×	Total
Malignant	1232	1390	1437	1370	5429
Benign	604	623	644	625	2496
Total	1836	2013	2081	1995	7925

**Table 3:** BreakHis dataset subtypes

Tumor subtype	40×	100×	200×	400×
AD	114	113	111	106
DU	864	903	896	788
FI	253	260	264	253
LO	156	170	163	137
MU	205	222	196	169
PA	145	142	135	138
PH	109	121	108	115
TU	149	150	140	130

Various models, such as the self-attention transformer ViT, ConvMixer, TokenLearner, and the proposed TokenMixer hybrid model, were used in this study. These models were applied to perform both (benign and malignant) and eight-class classification of BC histopathological subtype tissues.

### ViT

The self-attention ViT [16], was inspired by the original transformer model employed in natural language processing [44]. This mechanism operates by reshaping the input 2D image X ∈ R^*H*×*W* ×*C*^, where H represents height, W denotes width, and C signifies channels, into a sequence of non-overlapping image patches denoted as N_*xp*_ ∈ R^*N*×*P ·*2*C*^, where N is the number of patches and P representing the patch size given by

1 $$N=\frac{HW}{{P}^{2}}$$

Subsequently, the patches are flattened and mapped to D dimensions with a trainable linear projection, because the Transformer utilizes a constant latent vector size of D throughout its layers for classification [45]. In the self-attention function, the input patches are treated as a sequence of vectors. They first undergo a normalization step and are then processed by three linear layers: query (Q), key (K), and value (V). The attention output is obtained by calculating the dot product between Q and the transpose of K, which is then scaled down by a factor of d_*K*_, the square root of the dimension of K. Subsequently, this result is passed through a Softmax function. According to Eq. 1 for the ViT encoder, this process is repeated multiple times using multi-head attention. Here, Q, K, and V are projected multiple times (H times), where H represents the number of heads. Each projection simultaneously undergoes the self-attention mechanism. Finally, the outputs of all heads are concatenated to gather information from different representations of the same image.

2 $$attention \left(Q, K, V\right)=softmax \frac{{QK}^{T}}{\sqrt{dK}}V$$

The main process of the transformer encoder block employs a residual relationship that contains two elements: the tokenized input and the multi-head attention layer to apply the attention function. The output of the multi-head attention layer then under goes a two-step process. First, it’s normalized using layer normalization. Subsequently, it’s processed through a multilayer perceptron (MLP) head layer. Finally, the MLP head utilizes this output from the transformer encoder layers to create a probability distribution of labels, thereby enabling the prediction of the image class [26].

**Fig. 3:**
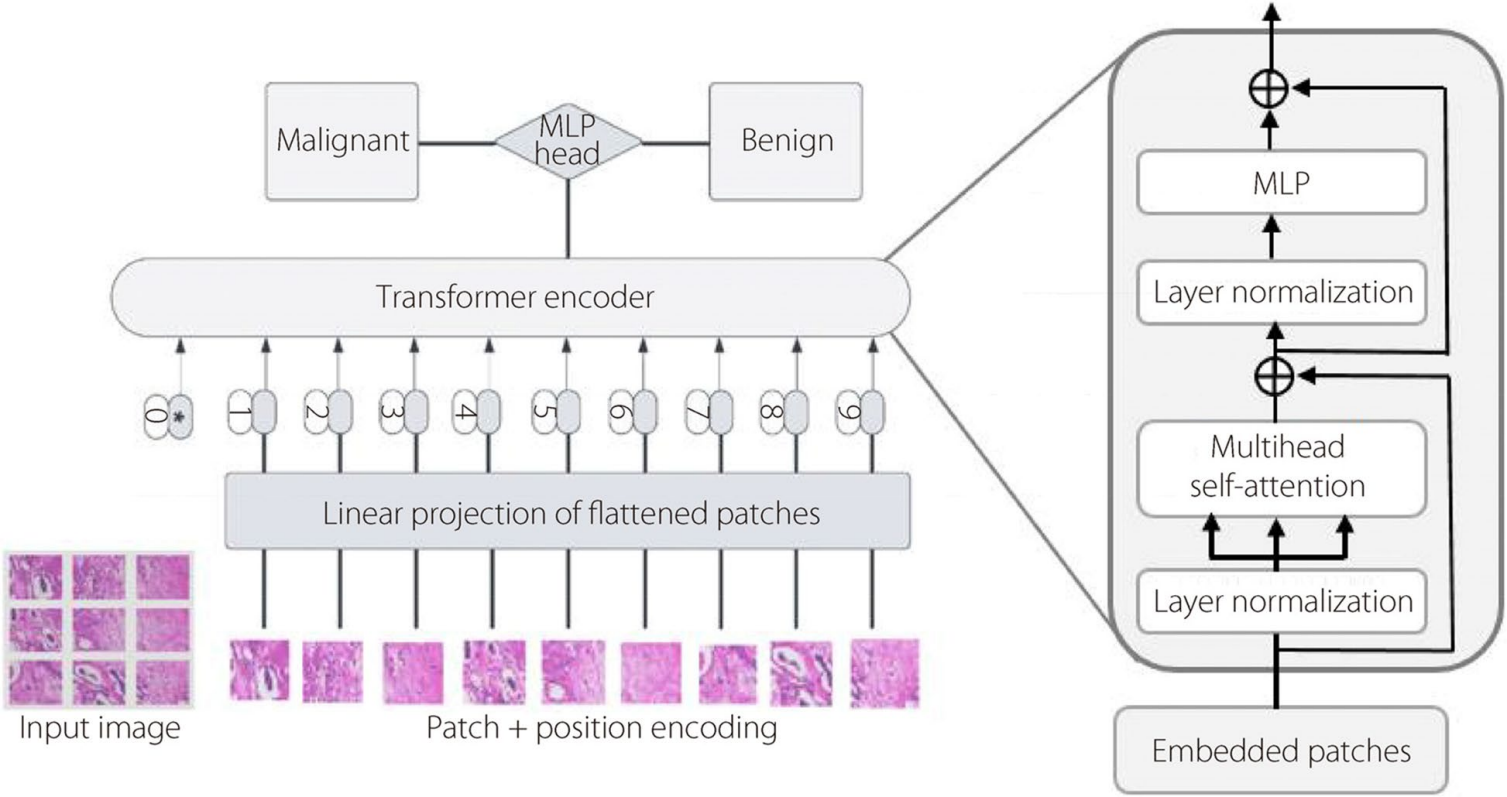
ViT self-attention architecture

The prevailing challenges involve the parameter count within ViT networks to enhance their performance. Figure 3 depicts the Transformer encoder architecture designed to classify histopathology images into benign or malignant categories. The process begins with a histopathology image partitioned into patches, with each patch encoded alongside positional information. These patches are then flattened and linearly projected into embeddings. The Transformer encoder comprises multi-head self-attention layers, enabling each patch to attend to others, thereby capturing the global dependencies. This is followed by layer normalization to stabilize the training process. The output from the encoder subsequently traverses an MLP multi-head equipped with an activation function to classify benign or malignant histopathology images. The specific parameters for the ViT model are listed in Table 4 and detailed in Algorithm 1. We chose a patch size of 14 × 14 for our implementation, which is slightly smaller than the 16 × 16 patches used in the original ViT research [16]. This choise was driven by our aim to enhance the capture of fine grained features within each tokenized input image and to enable more focused attention processes for each token. Such an approach is particularly effective for histopathological image analysis, where subtle details can play a crucial role in accurate diagnosis. When determining the number of layers, we looked at how to balance the models performance and complexity.

**Table 4:** Details of suggested ViT model parameters

Model	Image size	Patch size	Layers	Heads	Parameter
The suggested modified ViT	224 × 224	14	8	4	36,376,521

Our experiments led us to settle on an eight-layer model, the same experiment proposed by Abimouloud et al. [25] which we found struck an optimal balance between classification performance and computational requirements. Through this optimization, we arrived at a ViT model with 36,376,521 parameters. This parameter count represents a sweet spot, allowing for sufficient model expressivity while maintaining computational efficiency. This experimental approach demonstrates an efficient and potentially suitable method for real-world applications in medical image analysis.

**Figure:**
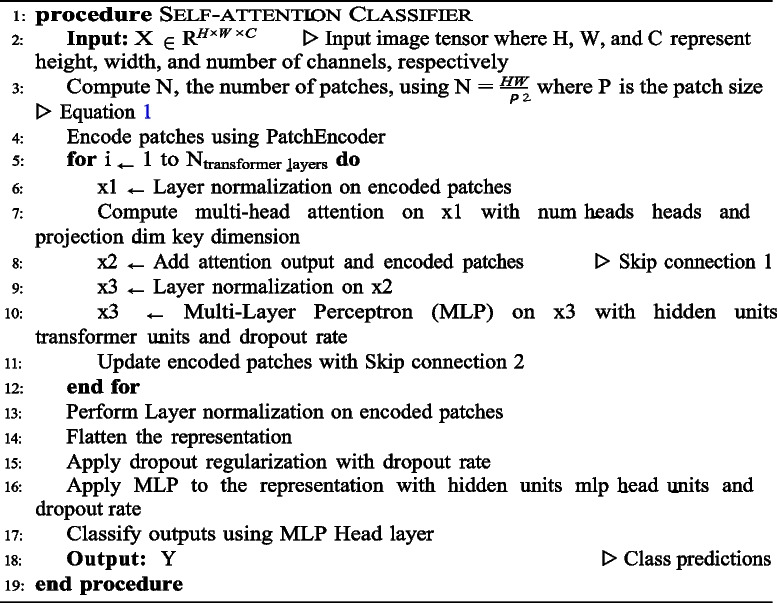
**Algorithm 1.** Self-attention mechanism

### TokenLearner

The self-attention mechanism obtained from Eq. 1 is acknowledged to have a high number of parameters and requiring a lot of processing resources. Consequently, performance improvements come at the cost of a large model size, requiring substantial computational GPU resources for model training. The TokenLearner model bridges this gap with the concept of tokenizing representations. Instead of working with fixed image patches, the attention maps of TokenLearner learn tokens dynamically, as outlined in Algorithm 2.

This approach allows TokenLearner to add significant regions to the input image, enabling the tokens to adjust to varying input data. Using a spatial attention mechanism, multiple spatial weight maps are calculated per frame, which are employed for the tokenization process. The objective of these attention maps is to discern crucial areas within the input. Each spatial weight map is then multiplied with the input to generate a token, which is processed by the subsequent learning modules. Moreover, TokenLearner can reduce the total number of image patches N while maintaining or even enhancing the classification accuracy. Simultaneously, this approach facilitates adaptive tokenization, allowing tokens to be selected dynamically selected based on the image patches. The tokenization mechanism steps are described as follows [46]:

**Figure:**
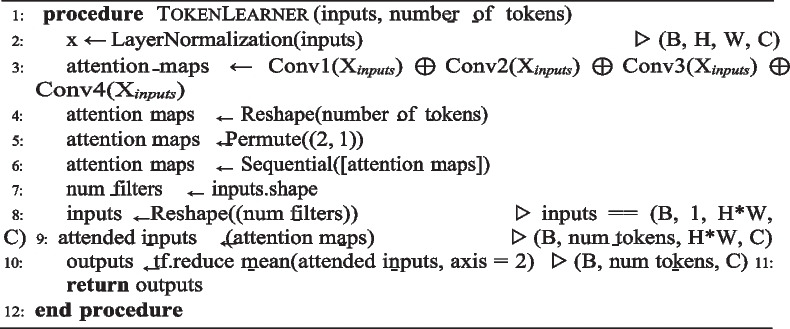
**Algorithm 2.** Attention maps TokenLearner algorithm

**Input tensor representation:** The input with a space-time shape present as X ∈ R^*T* ×*H*×*W* ×*C*^, X_*t*_ be a temporal slice of it, corresponding to frame t: X_*t*_∈ R^*H*×*W* ×*C*^.

**Token generation for each time frame:** In each time frame t, generate a series of S tokens Z_*t*_ = [z_*i*_]^*S*^ is generated from the input frame X_*t*_. To learn a tokenizer function, z_*i*_ = A_*i*_(X_*t*_) mapping X_*t*_ to a token vector z_*i*_ ∈ R^*H*×*W* ×*C*^ → R^*C*^. The tokenizer function A_*i*_ is learned to adaptively select informative spatial locations in X_*t*_, resulting in a set of adaptively changing spatial selections.

**Implementation of tokenizer with spatial attention:** The tokenizer function, denoted as z_*i*_ = A_*i*_(X_*t*_) operates using a spatial attention mechanism. It assesses the significance of various regions in the input image represented by X_*t*_, by generating a weight map of size H × W for each region. This weight map α_*i*_(X_*t*_) is a function generating the spatial H × W × 1 weight map. These weight maps are then multiplied with the input image X_*t*_ to create tokens denoted as z_*i*_ generated by Eq. 3.

3 $${Z}_{i}={A}_{i}\left({X}_{t}\right)=\rho \left({X}_{t}\odot {A}_{iw}\right)=\rho \left({X}_{t}\odot \gamma ({\alpha }_{i}\left({X}_{t}\right))\right)$$

where ⊙ is the Hadamard product and A_*iw*_ ∈ R^*H*×*W* ×*C*^ is an intermediate weight tensor computed with the function α_*i*_(X_*t*_) and the broadcasting function γ().

**Fig. 4:**
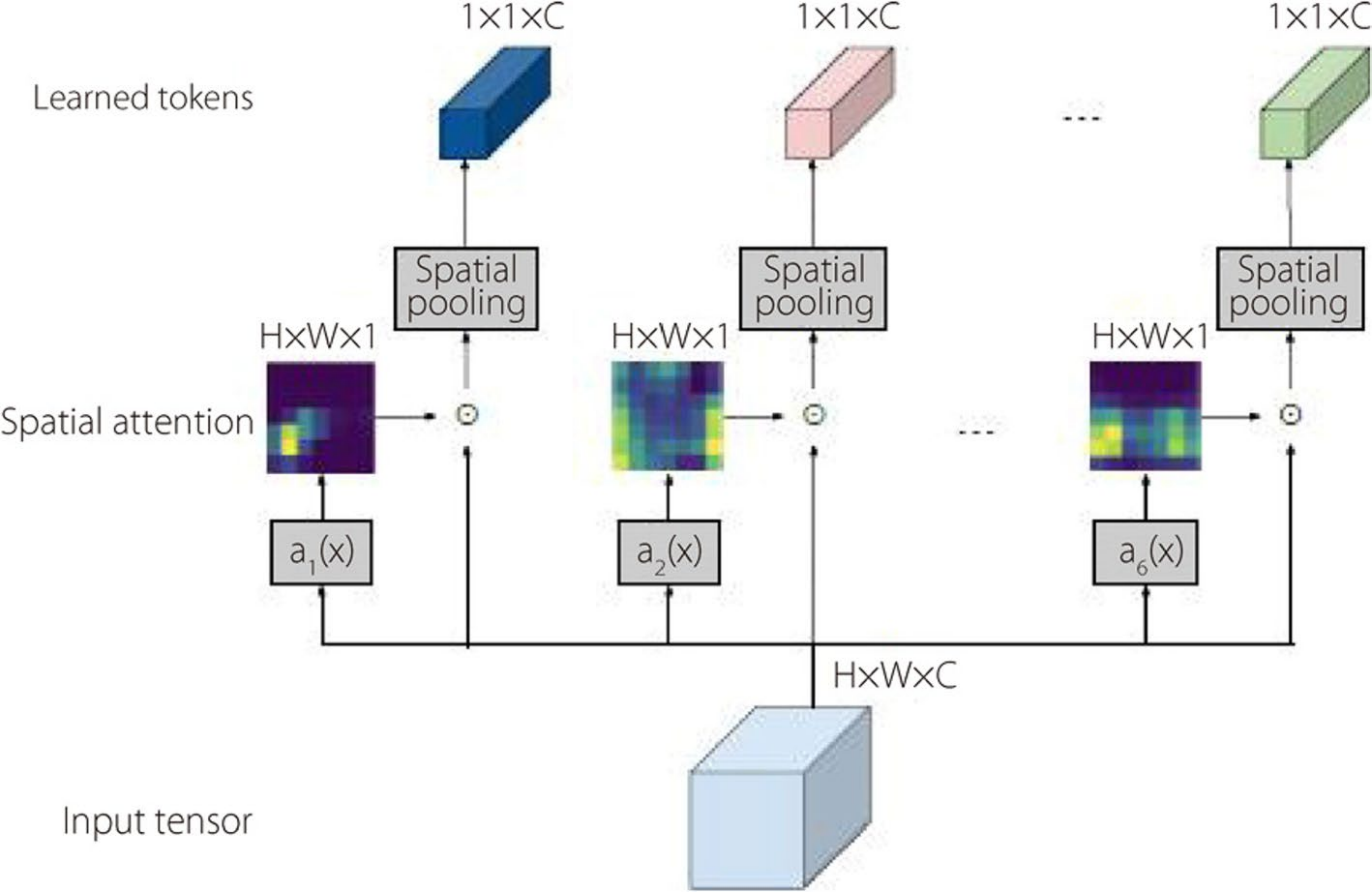
The token vector generation from the input patches (courtesy: [46])

As demonstrated in Fig. 4, spatial global average pooling is applied to reduce the dimensionality to R^*C*^.

**Output tensor and self-attention:** The resulting tokens are gathered to form the output tensor Z_*t*_ = [z_*i*_]^*S*^ ∈ R. The overall process resembles element-wise spatial self-attention Z = Z_*t*_. The learned tokens from TokenLearner are provided to subsequent layers for multi-head self-attention used in transformer encoder block. This reduces computations for subsequent layers, as they only need to process a small number of tokens, significantly reducing computational complexity.

### ConvMixer

The ConvMixer [47] architecture operates directly on the patches, ensuring consistent resolution and size representation across all layers. This prevents downsampling of the representation in successive layers. Notably, it segregates “channel-wise mixing” from the spatial mixing of information, introducing a distinct separation in handling different aspects of image patches. This architecture comprises a patch-embedding layer with a residual connection between fully convolutional layers. Specifically, patch embeddings are implemented using the following parameters: patch size (p), embedding dimension (h), cin input channels, h output channels, a kernel size of (p), and a stride of (p). This implementation is depicted in Eq. 4.

4 $${Z}_{0}=BN(\sigma \left(Convcin \to h\left(X, stride=p, kernel size=p\right)\right))$$

The ConvMixer block, as depicted in Algorithm 3, is constructed using a combination of depth-wise convolution layers, followed by a point-wise convolution with a kernel size of 1 × 1. Following multiple applications of the ConvMixer block, a global pooling operation is carried out to obtain a feature vector of size h. The resulting feature vector is then forwarded to a Softmax function for final classification. The Gaussian Error Linear Uni (GELU) activation function is applied after each convolution operation. Two distinct types of convolutional layers are employed within the ConvMixer architecture. First, depth-wise convolution equations are used to intertwine the spatial locations within the images, as shown in Eq. 5. Second, point-wise convolution equations are utilized to mix the channel-wise information across the patches. The incorporation of larger kernel sizes facilitates a broader receptive field, as shown in Eq. 6, contributing to enhanced feature extraction and representation.

**Figure:**
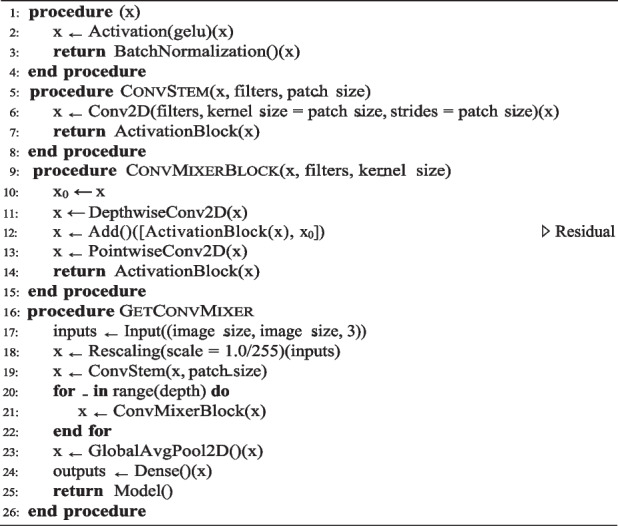
**Algorithm 3.** ConvMixer model

5 $$Z_l^\prime=BN\left(\sigma\left(ConvDepthwise\left(Z_{l-1}\right)\right)+Z_{l-1}\right)$$

6 $$Z_{l+1}=BN(\sigma(ConvPointwise(Z_l^\prime)))$$

**Fig. 5:**
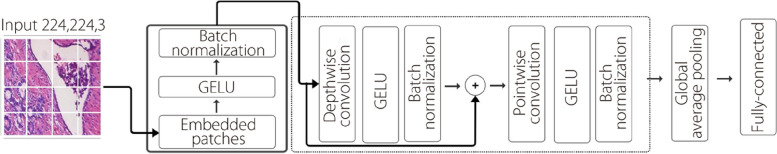
Convmixer classification architecture

The ConvMixer architecture shown in Fig. 5 can be broken down into the following stages:

- Input: The input to ConvMixer is a histopathological image, which is typically represented as a 3D tensor of numbers. The first dimension corresponds to the height of the image, the second to the width of the image, and the third to the number of color channels in the image (three for RGB images). In the specific case of the diagram, the input has a dimension of 224 × 224 × 3 (height, width, and RGB channels).
- Batch normalization: This technique is often used in deep neural networks to improve the stability of the training process. The convolution operation systematically moves a learnable filter across an input image. This filter performs local computations to detect specific patterns or features at each position. The result is a feature map that captures the presence and intensity of these detected features throughout the image.
- GELU: The GELU is a type of activation function that is often used in deep neural networks. Activation functions are used to introduce nonlinearity into the network, which allows it to learn more complex patterns in the data.
- Point-wise convolution: This type of convolution operation uses a 1 × 1 filter. It is often used to reduce the dimensionality of the feature maps or project them into a higher-dimensional space.
- Global average pooling: This operation averages the elements of the feature maps to generate a single vector. This vector can then be used to classify an image or predict some other property of the image.
- Fully connected layer: This layer combines the features extracted from the convolutional layers into a single output vector. The output vector is then used to classify the image or predict some other property of the image.

### TokenMixer

TokenLearner demonstrates proficient generation of the weight map α_*i*_(X_*t*_) from the input patches; however, further enhancement is required in tokenizing the extracted features from the images outlined in Eq. 3. TokenMixer addresses this gap by refining the processing of these patches. Initially, the inputs are normalized, which is followed by applying depth-wise convolution and batch normalization layers to each patch sequentially. This sequence facilitates the integration of channel-wise information across the patches, thereby enhancing the feature extraction and representation. Subsequently, four convolutional layers are employed to generate the weight map α_*i*_(X_*t*_), with each layer focusing on different segments of the input. The resulting weight maps undergo element-wise multiplication with the input, and the outcomes are aggregated by pooling. This output features a substantially reduced number of generated tokens z_*i*_. The technique used to generate the weight maps is shown in Algorithm 4.

**Fig. 6:**
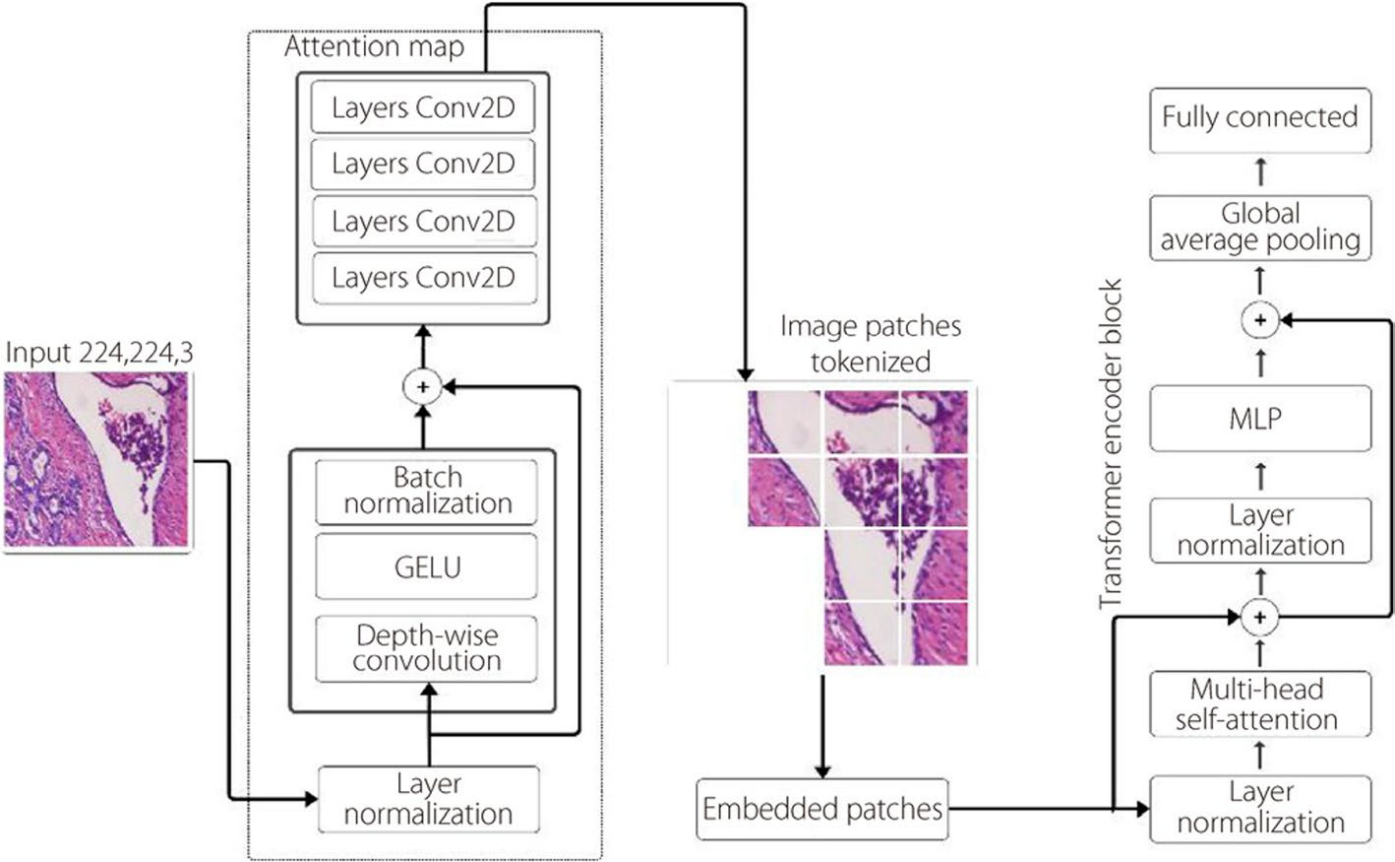
TokenMixer architecture

The use of ConvMixer blocks based on Eqs. 4 and 5 with multiple convolution layers plays a pivotal role in enhancing the expressivity, whereas the introduction of spatial attention through the attention maps helps to preserve pertinent information from the inputs and prevent overfitting. Both components are integral to the functionality of TokenMixer, particularly in situations in which a significant reduction in the number of patches is employed. The output selected patches Z_*t*_ are then transferred to a Transformer block to capture the long-range dependencies and enhance the feature representations. Layer normalization is applied, multi-head self-attention is employed to capture the global context, and layer normalization and global average pooling are used for the final encoded patches. Finally, the MLP head is typically the output layer, which contains as many neurons as the classes in the classification task. Each neuron in the output layer corresponds to a specific class, the sigmoid activation function classifies the final representation in binary and the Softmax activation function is used for multi-classification, as detailed in Algorithm 5. The methodology incorporating attention map layers, as illustrated in Fig. 6, is tailored to focus on crucial regions of the input images.

1. Input layer: Receives images with dimensions of 224 × 224 × 3 (height, width, and RGB channels).
2. Preprocessing: Involves normalization or resizing to ensure consistent image formats.
3. Patch extraction:
  Depth-wise separable convolution layer: Extracts spatial features.
  Convolution layers: Generate the attention map for further feature extraction to minimize the number of input patches.
  Normalization layers: Stabilize training by normalizing activations.
  GELU activation function: Introduces nonlinearity for intricate pattern learning.
4. Patch embedding: Transforms extracted patches into higher-dimensional vectors for enhanced representation.
5. Transformer encoder blocks: These blocks include:
  Multi-head layer: Allows simultaneous attention to various parts of the image, capturing the relationships and dependencies between toknes.
  Global average pooling: Averages elements across feature maps, producing a single vector encapsulating significant features from the entire image.
  Fully connected layer: Maps the output vector from global average pooling to class scores, representing the probabilities for different categories of tumor classification tasks.

In summary, the architecture combines TokenMixer with Transformer encoder blocks and leverages attention map layers to prioritize image regions and capture interpatch relationships. This approach outperforms TokenLearner in histopathological image classification.

**Figure:**
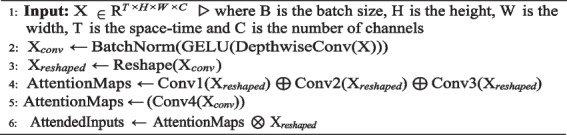
**Algorithm 4.** Attention maps TokenMixer algorithm

**Figure:**
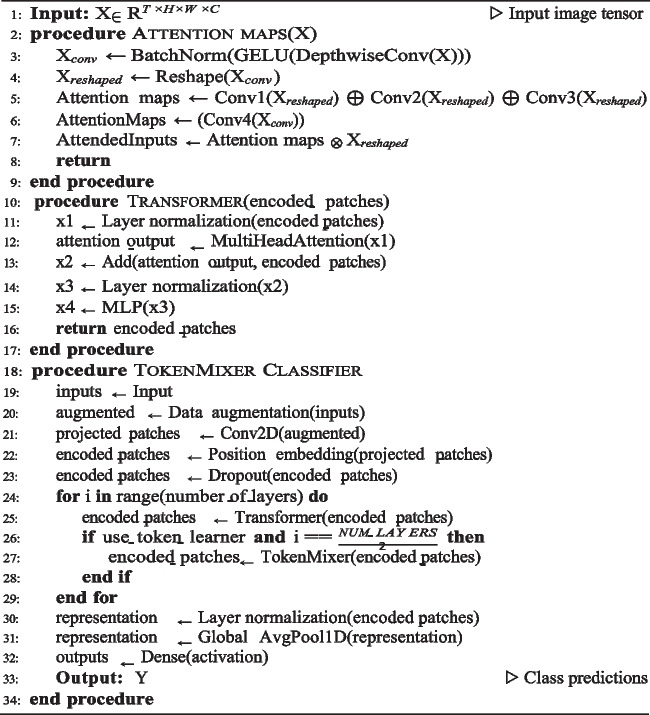
**Algorithm 5.** Global algorithm

## Results

### Dataset splitting

The study goal is to diagnose BC efficiently and accurately using a dataset consisting of 7925 microscopic biopsy images, which were categorized into four magnification levels: 40×, 100×, 200×, and 400×. In the binary classification task, the images were divided into two classes to distinguish between malignant and benign tumors at each magnification level. For the multi-classification task, images were classified into eight tumor subtypes at each magnification. The dataset underwent an 80-20 split, with 80% allocated for training and 20% for testing. Further division was implemented within the training set, with 80% for the training process and 20% for validation to prevent overfitting. We utilized a HP Z8 G4 workstation to develop and implement our systems.

- Memory (RAM): 96.00 GB
- Processor: Intel(R) Xeon(R) Silver 4108 CPU @ 1. 80 GHz 1. 80 GHz.
- Graphic Processing Unit: GeForce RTX 2080 Ti, GeForce RTX 3090
- System type: 64-bit operating system, × 64 processor. We utilized Python 3.11.

Subsequently, all codes executions were carried out utilizing the execution environments provided by Google Colab Pro.

### Dataset preprocessing

Balancing datasets is crucial for developing an impartial and performant classification model. The consequences of an imbalanced dataset include poor generalizability and an increased risk of misclassification, which pose a common challenge in the medical field. To address the imbalance present in the BreakHis dataset, data augmentation was employed as a strategy to alleviate data scarcity, and the hyperparameters used in this technique are detailed in Table 5. In addition, threefold cross-validation was adopted for all models in the multi-classification training task. To ensure a fair comparison, all models underwent the same steps of data preprocessing and threefold cross-validation and utilized the same hyperparameter values in the training setups. The hyperparameter values are listed in Table 6.

**Table 5:** Data augmentation parameters

Data augmentation technique	Value
Rotation range	5
Width shift range	0.1
Height shift range	0.1
Zoom range	0.001
Fill mode	0.001

**Table 6:** Hyperparameter settings

Hyperparameter	Value
Batch size	32
Number of epochs	100
Optimizer	Adam
Loss function	Binary Crossentropy, categorical Crossentropy

### Evaluation metrics

To assess the efficacy of our systems, we employed a comprehensive set of evaluation metrics. These included accuracy, precision, sensitivity, specificity, area under the curve (AUC), F1 score, Matthews correlation coefficient (MCC), kappa statistic, and G-mean. Each of these metrics was calculated based on the confusion matrix generated by our system’s performance. In our analysis, the confusion matrix providing nuanced insights into the classification outcomes. It enabled us to differentiate between several crucial categories: true positives (TP), which are correctly identified malignant BC tumors; true negatives (TN), representing accurately classified benign BC tumors; false positives (FP), where benign tumors were erroneously labeled as malignant; and false negatives (FN), cases where malignant tumors were mistakenly categorized as benign. The equations for these performance metrics are as follows:

7 $$\text{Accuracy }\left(\%\right)=\frac{\text{TP}+\text{TN}}{\textrm{TP}+\textrm{TN}+\textrm{FP}+\textrm{FN}}$$

8 $$\text{Sensitivity}\left({\%}\right)=\frac{\text{TP}}{\textrm{TP}+\textrm{FN}}$$

9 $$\text{Precision}\left({\%}\right)=\frac{\text{TP}}{\textrm{TP}+\textrm{FP}}$$

10 $$\text{Specificity}\left({\%}\right)=\frac{\text{TN}}{\textrm{TN}+\textrm{FP}}$$

11 $$\text{F }1\text{score}({\%}) =\frac{2 \times [\text{Precision }\times \text{ Recall}]}{\textrm{Precision }+\textrm{ Recall}}$$

12 $$\text{AUC}\left({\%}\right)=\frac{\text{Sensitivity}}{\textrm{Specificity}}$$

13 $$\text{MCC }= \frac{\text{T P }\times \text{ T N }-\text{ FP }\times \text{ FN}}{\sqrt{(\textrm{T P }+\textrm{ FP})(\textrm{T P }+\textrm{ FN})(\textrm{T N }+\textrm{ FP})(\textrm{T N }+\textrm{ FN})}}$$

14 $$\upkappa =\frac{\text{Po }-\text{ Pe}}{1 -\textrm{ Pe}}$$

15 $$G=\sqrt{\frac{\text{precision }\times \text{ recall}}{\textrm{precision }+\textrm{ recall}}}$$

16 $$\text{Total}_{-}\text{Training}_{-}\text{Time}(\text{S}) =\text{ end}_{-}\text{time}-\text{start}_{-}\text{time}$$

17 $$\text{Avg}_{-}\text{Training}_{-}\text{Time}_{-}\text{per}_{-}\text{epoch}=\frac{{\text{Total}}_{-}{\text{Training}}_{-}\text{Time}}{{\textrm{Num}}_{-}\textrm{epochs}}$$

## Results

This subsection presents a thorough evaluation performed through various experiments of BC classification into binary and multi-classification conducted using the proposed models.

### BC binary classification

The proposed model achieved excellent performance in the binary classification of benign and malignant classes, as presented in Table 7. The models exhibited high scores in all evaluation metrics calculated across various magnification levels using the BreakHis dataset. These results provide robust evidence that the hybrid ViT-CNN models enhances DL approaches for the analysis of BC histopathology images.

**Table 7:** Binary classification performance metrics for the different models at different magnification levels

Model	Magnification	Accuracy	Precision	Sensitivity	Specificity	AUC	F1 score
ConvMixer	400*×*	96.74%	96.49%	96.09%	96.09%	99.52%	96.29%
	200*×*	95.53%	94.31%	95.44%	95.44%	99.46%	94.84%
	**100***×*	**98.60**%	98.57%	98.18%	98.18%	99.79%	98.37%
	40*×*	97.52%	97.68%	97.41%	97.41%	99.85%	97.50%
TokenLearner	400*×*	93.48%	92.12%	93.45%	93.45%	98.41%	92.73%
	**200*×***	**95.53**%	95.15%	95.15%	94.34%	99.49%	94.73%
	100*×*	92.31%	91.46%	90.62%	90.62%	97.56%	91.02%
	40*×*	93.32%	93.49%	93.19%	93.19%	98.13%	93.28%
ViT	400*×*	95.38%	94.85%	94.66%	94.66%	99.16%	94.75%
	**200*×***	**96.53**%	96.55%	95.28%	95.28%	99.38%	95.88%
	100*×*	95.10%	93.88%	95.03%	95.03%	98.10%	94.42%
	40*×*	93.70%	94.34%	9345%	93.45%	99.58%	93.64%
TokenMixer	400*×*	95.11%	94.15%	94.88%	94.88%	98.63%	94.50%
	**200*×***	**97.02**%	96.33%	96.74%	96.74%	99.70%	96.53%
	100*×*	94.64%	94.21%	93.31%	93.31%	98.88%	93.74%
	40*×*	92.75%	93.05%	92.57%	92.57%	98.32%	92.70%

A comparison of the best performance of the systems is presented in Table 8. Notably, the ConvMixer model at 100× magnification exhibited the highest accuracy, with an impressive 98.60%, a precision of 98.57%, a sensitivity of 98.18%, a specificity of 98.18%, AUC of 99.79%, and an F1 score of 98.37%. In addition, the TokenLearner model exhibited outstanding performance at 200× magnification, achieving an accuracy of 95.53%, with precision, sensitivity, specificity, AUC, and F1 score of 95.15%, 95.15%, 94.34%, 99.49%, and 94.73%, respectively. The ViT model exhibited superior performance at 200× magnification, achieving an accuracy of 96.53%, with precision, sensitivity, specificity, AUC, and F1 score of 96.55%, 95.28%, 95.28%, 99.38%, and 95.88%, respectively. Furthermore, the proposed TokenMixer model exhibited excellent accuracy at 200× magnification, with an accuracy of 97.02%. The precision, sensitivity, specificity, AUC, and F1 score were 96.33%, 96.74%, 96.74%, 99.70%, and 96.53%, respectively. Notably, the TokenMixer model achieved a short training time of 391.71 s, compared with the ConvMixer model at 2729.14 s and the ViT model at 639.66 s.

**Table 8:** The best performance in binary classification for each model

Model	ConvMixer	Tokenlearner	ViT	TokenMixer
Total training time (s)	2729.14	391.29	639.66	391.71
Average training time per epoch (s)	27.29	4.34	6.39	4.35
Accuracy	**98.60%**	**95.53%**	**96.53%**	**97.02%**
Precision	98.57%	95.15%	96.55%	96.33%
Sensitivity	98.18%	95.15%	95.28%	96.74%
Specificity	98.18%	94.34%	95.28%	96.74%
AUC	99.79%	99.49%	99.38%	99.70%
F1 score	98.37%	94.73%	95.88%	96.53%
MCC	0.8367	0.8947	0.9417	0.9065
Kappa	83.59%	89.37%	94.14%	90.59%
G-mean	90.99%	93.80%	96.63%	94.63%
Best magnification	100×	200×	200×	200×
Total parameter	577,282	1,402,169	36,376,521	1,403,961

**Fig. 7:**
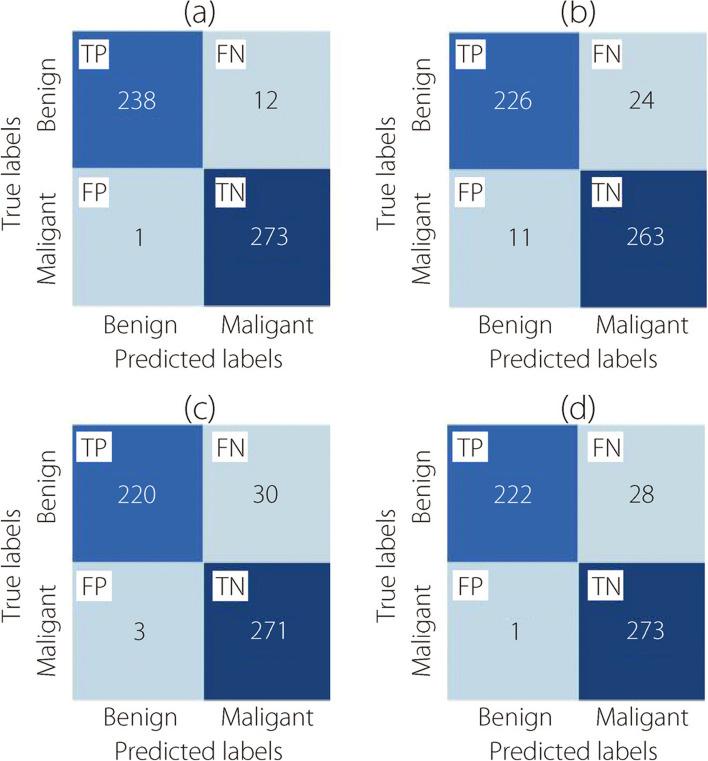
The binary classification confusion matrices at 40× magnification for (**a**) ConvMixer, (**b**) TokenLearner, (**c**) ViT, and (**d**) TokenMixer models

**Fig. 8:**
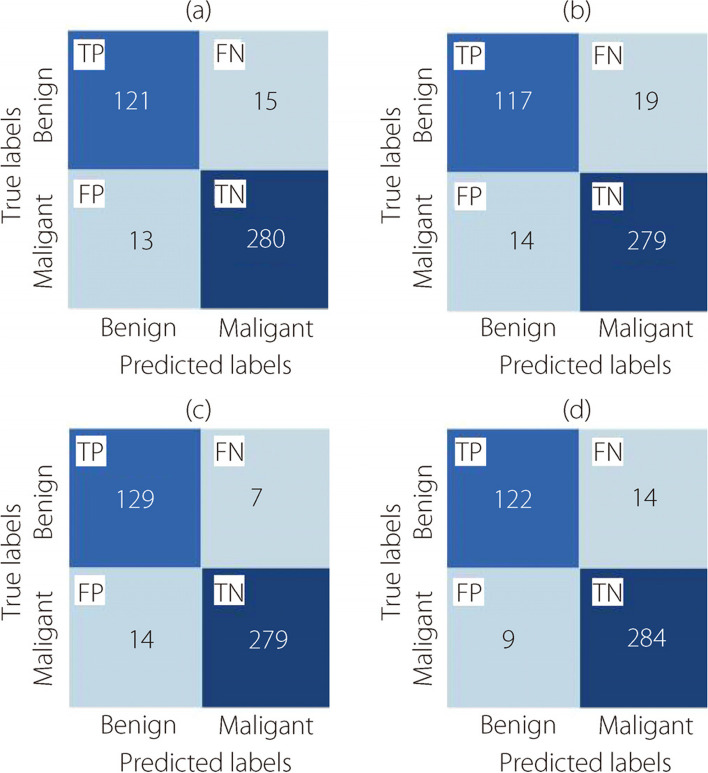
The binary classification confusion matrices at 100× magnification for (**a**) ConvMixer, (**b**) TokenLearner, (**c**) ViT, and (**d**) TokenMixer models

**Fig. 9:**
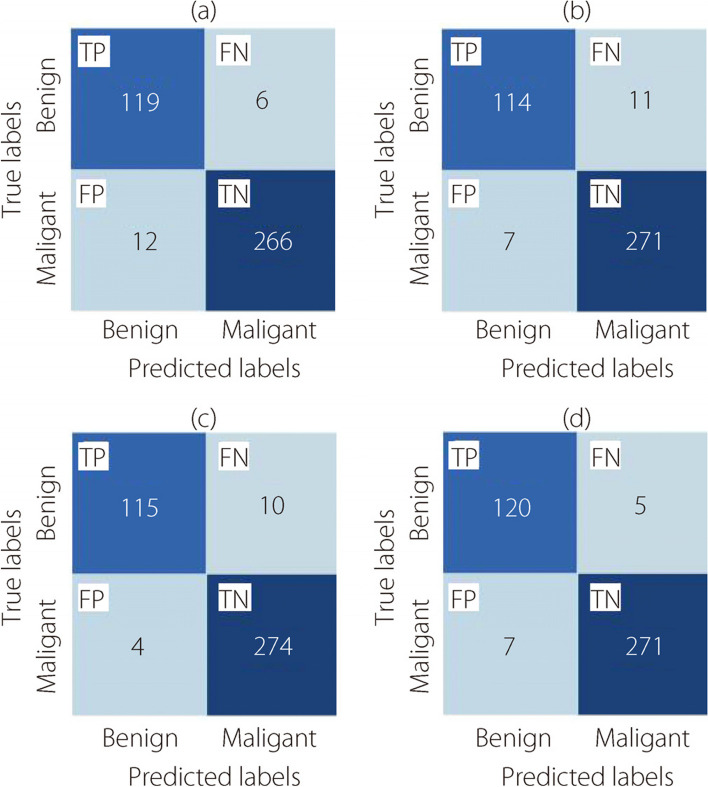
The binary classification confusion matrices at 200× magnification for (**a**) ConvMixer, (**b**) TokenLearner, (**c**) ViT, and (**d**) TokenMixer models

**Fig. 10:**
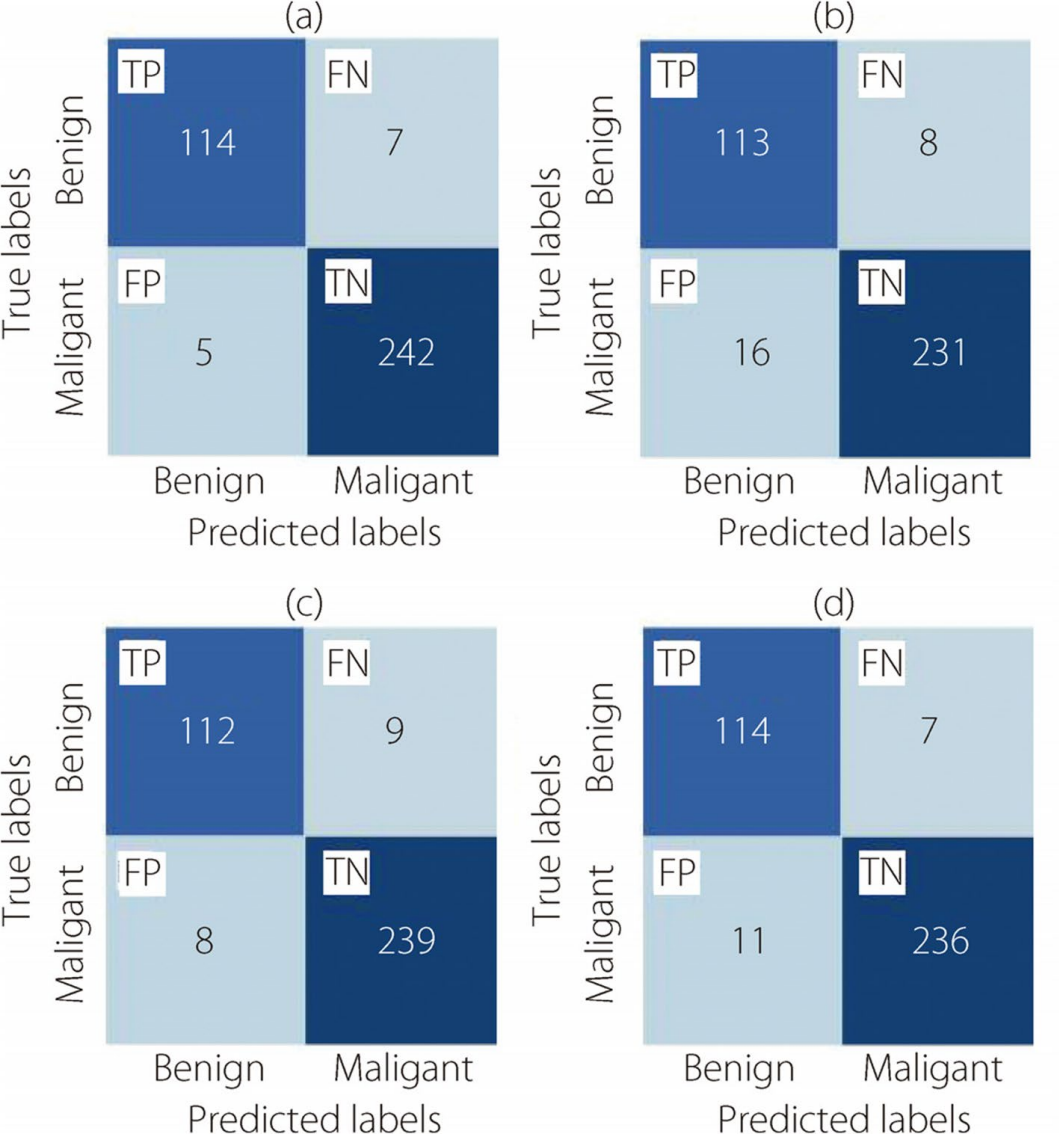
The binary classification confusion matrices at 400× magnification for (**a**) ConvMixer, (**b**) TokenLearner, (**c**) ViT, and (**d**) TokenMixer models

As introduced in Figs. 7, 8, 9, and 10, the confusion matrices for the binary classification task were generated under the 40×, 100×, 200×, and 400× levels of magnification, respectively.

Figure 8 illustrates the confusion matrices for the two-class classification task. The evaluations were conducted at a magnification of 100×.

The confusion matrices presented in Fig. 9 show the results of the classification task at a magnification of 200×.

Figure 10 shows the confusion matrices generated for the classification task using the Brekhis image dataset at a magnification of 400×.

**Fig. 11:**
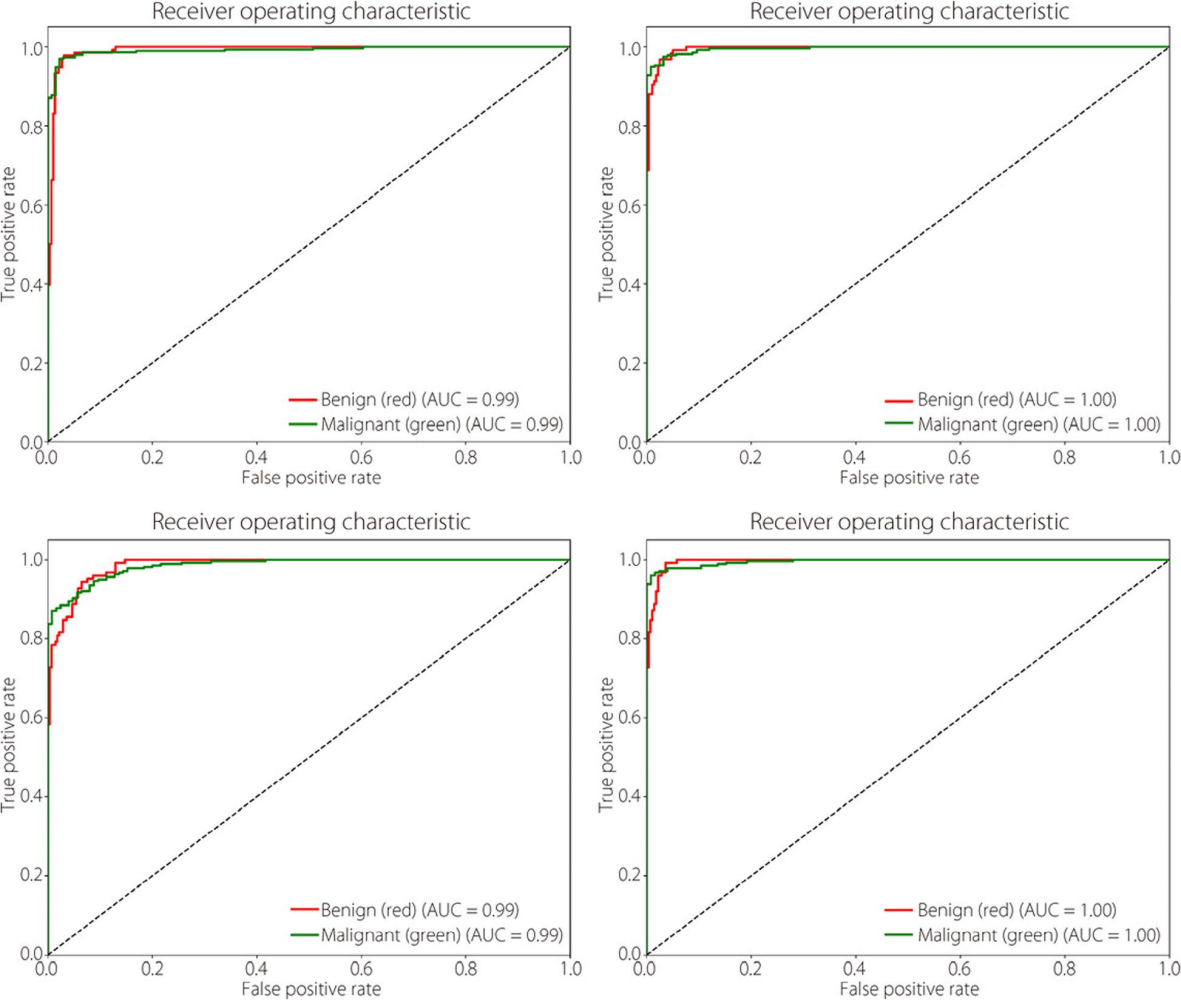
The binary classification ROC curves for (**a**) ConvMixer, (**b**) TokenLearner, (**c**) ViT, and (**d**) TokenMixer models

The ROC curves for each models optimal performance in classifying benign and malignant tumours are shown in Fig. 11. Notably, the proposed TokenMixer model exhibited substantial improvements in the classification accuracy.

### BC multi-classification

Since binary classification has more samples than multi-classification, deep learning models are facing increasing challenges in this area. For effective training and precise predictions, these challenges require larger datasets. Our study’s primary goal was to assess the models’ ability to enhance the classification performance of eight different subtypes of BC tumours in response to these changes. The study also thoroughly examined how the proposed TokenMixer model performed in classifying these eight BC subtypes across various magnification levels. To ensure a robust and reliable assessment across different magnifications, we used a three-fold cross-validation technique. Table 9 presents the best performance results of our proposed systems. As shown in Table 10, our proposed systems performed exceptionally well in multi-classification tasks, based on the evaluation metrics we used.

**Table 9:** The classification metric values for eight BC subtypes across different models

	Magnification	Accuracy	Precision	Sensitivity	Specificity	AUC	F1 score
ConvMixer	400×	92.70%	93.06%	92.70%	98.77%	97.66%	92.67%
	**200**×	**94.71**%	94.77%	94.71%	99.06%	99.44%	94.68%
	100×	94.10%	94.21%	94.10%	98.92%	99.54%	94.04%
	40×	94.67%	94.81%	94.67%	99.05%	99.47%	94.53%
TokenLearner	400×	89.57%	90.40%	89.57%	98.34%	99.04%	89.61%
	200×	89.30%	89.84%	89.30%	98.27%	98.61%	89.31%
	100×	90.55%	90.85%	90.55%	98.44%	98.39%	90.38%
	**40**×	**92.41**%	92.80%	92.41%	98.75%	99.19%	92.39%
ViT	400×	89.84%	90.01%	89.84%	98.36%	93.36%	89.78%
	200×	91.67%	91.62%	91.67%	98.54%	97.50%	91.32%
	100×	91.46%	91.64%	91.46%	98.60%	95.77%	91.43%
	**40**×	**93.67**%	93.74%	93.67%	98.86%	95.37%	93.53%
TokenMixer	400×	90.93%	91.02%	90.93%	98.51%	98.86%	90.80%
	200×	91.67%	90.72%	90.61%	98.41%	99.02%	90.55%
	100×	90.73%	91.10%	90.73%	98.45%	98.94%	90.53%
	**40**×	**93.29**%	93.43%	93.29%	98.84%	99.23%	93.26%

**Table 10:** The best multi-classssification values for each model

Model	ConvMixer	TokenLearner	ViT	TokenMixer
Total training time (s)	6436.47	1175.04	1730.19	1173.56
Average training time per epoch (s)	64.36	11.75	17.30	11.73
Accuracy	**94.71**%	**92.41**%	**93.67**%	**93.29**%
Precision	94.77%	92.80%	93.74%	93.43%
Sensitivity	94.71%	92.41%	93.67%	93.29%
Specificity	99.06%	98.75%	98.86%	98.84%
AUC	99.44%	99.19%	95.37%	99.23%
F1 score	94.68%	92.39%	93.53%	93.26%
MCC	0.9307	0.9028	0.904	0.8659
Kappa	93.04%	90.26%	90.32%	86.53%
G-mean	94%	92%	91%	89%
Best magnification	200×	40×	40×	40×
Total parameter	577,282	1,402,169	36,376,521	1,403,961

**Fig. 12:**
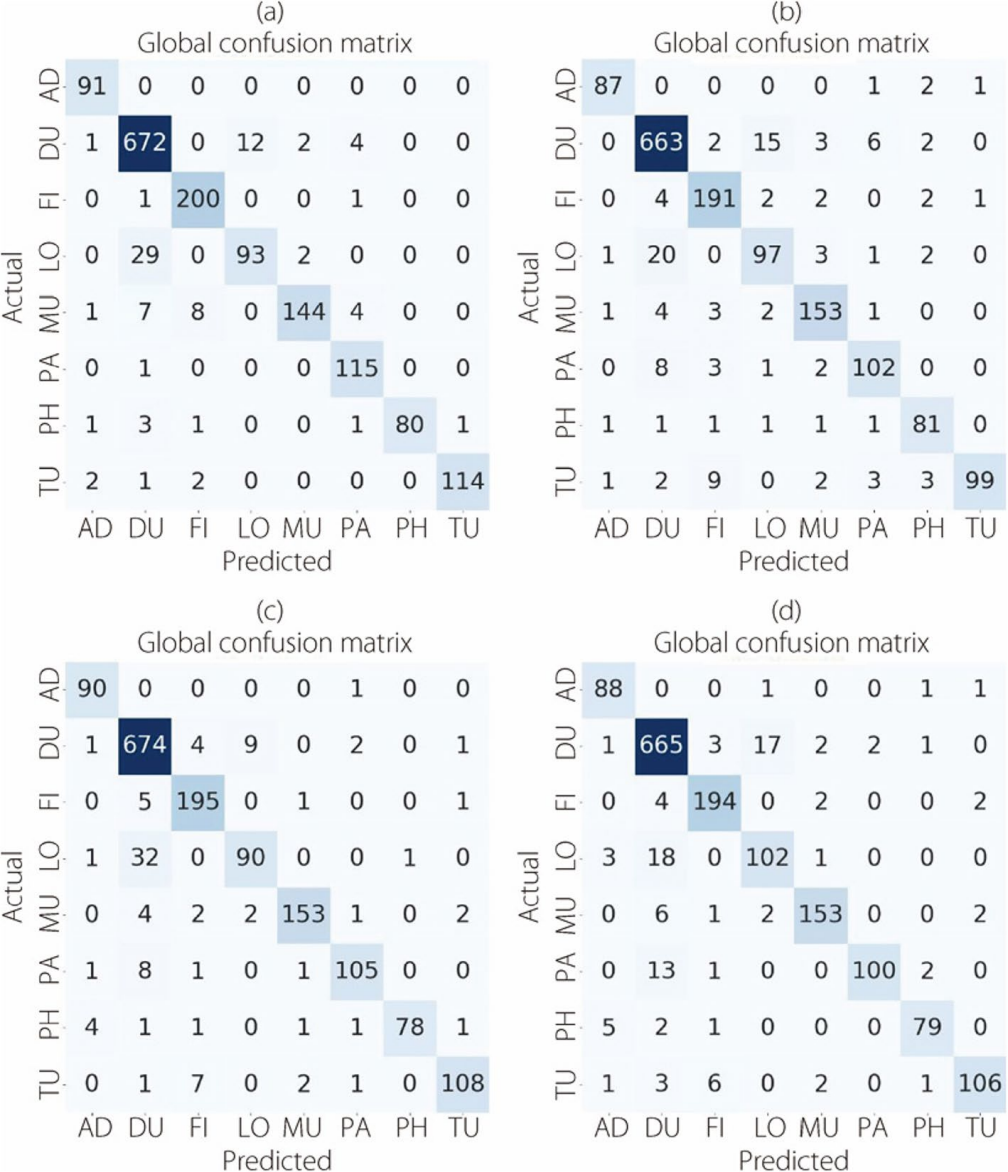
The multi-classification confusion matrices at 40× magnification for (**a**) ConvMixer, (**b**) TokenLearner, (**c**) ViT, and (**d**) TokenMixer models

Figure 12 shows the confusion matrices for the classification of multi-BC subtypes, focusing on the 40× level of magnification.

**Fig. 13:**
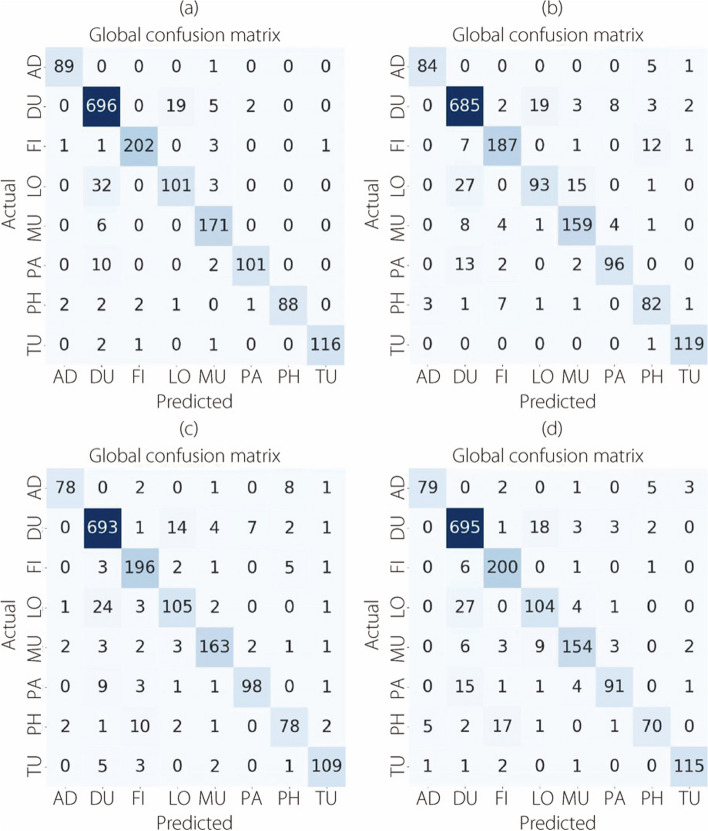
The multi-classification confusion matrices at 100× magnification for (**a**) ConvMixer, (**b**) TokenLearner, (**c**) ViT, and (**d**) TokenMixer models

Figure 13 presents the confusion matrices resulting from the multi-classification task on the BreakHis dataset at a magnification level of 100×.

**Fig. 14:**
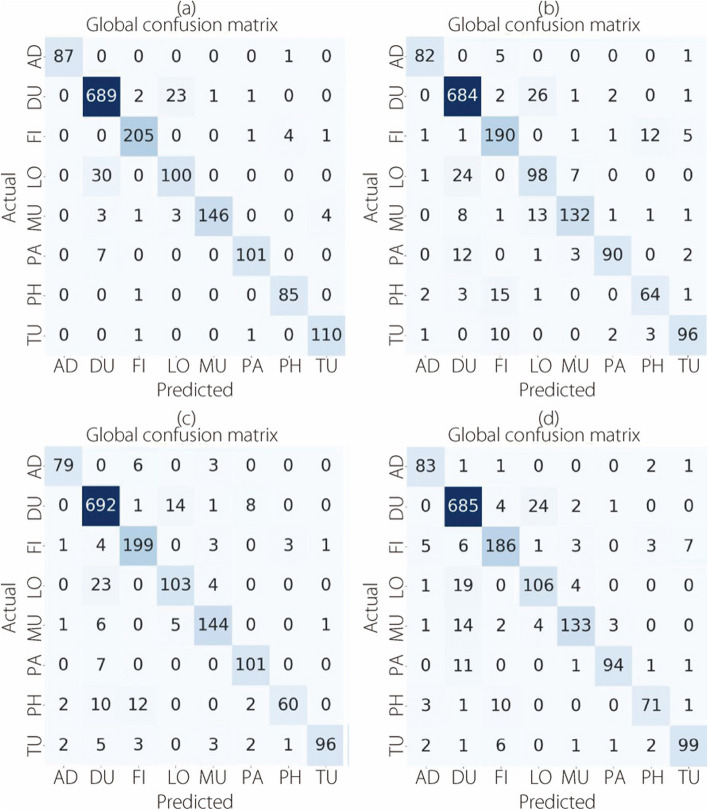
The multi-classification confusion matrices at 200× magnification for (**a**) ConvMixer, (**b**) TokenLearner, (**c**) ViT, and (**d**) TokenMixer models

The confusion matrices presented in Fig. 14 provide a visual representation of the outcomes of the multi-classification task. These evaluations were conducted using the BreakHis dataset at a magnification level of 200×.

**Fig. 15:**
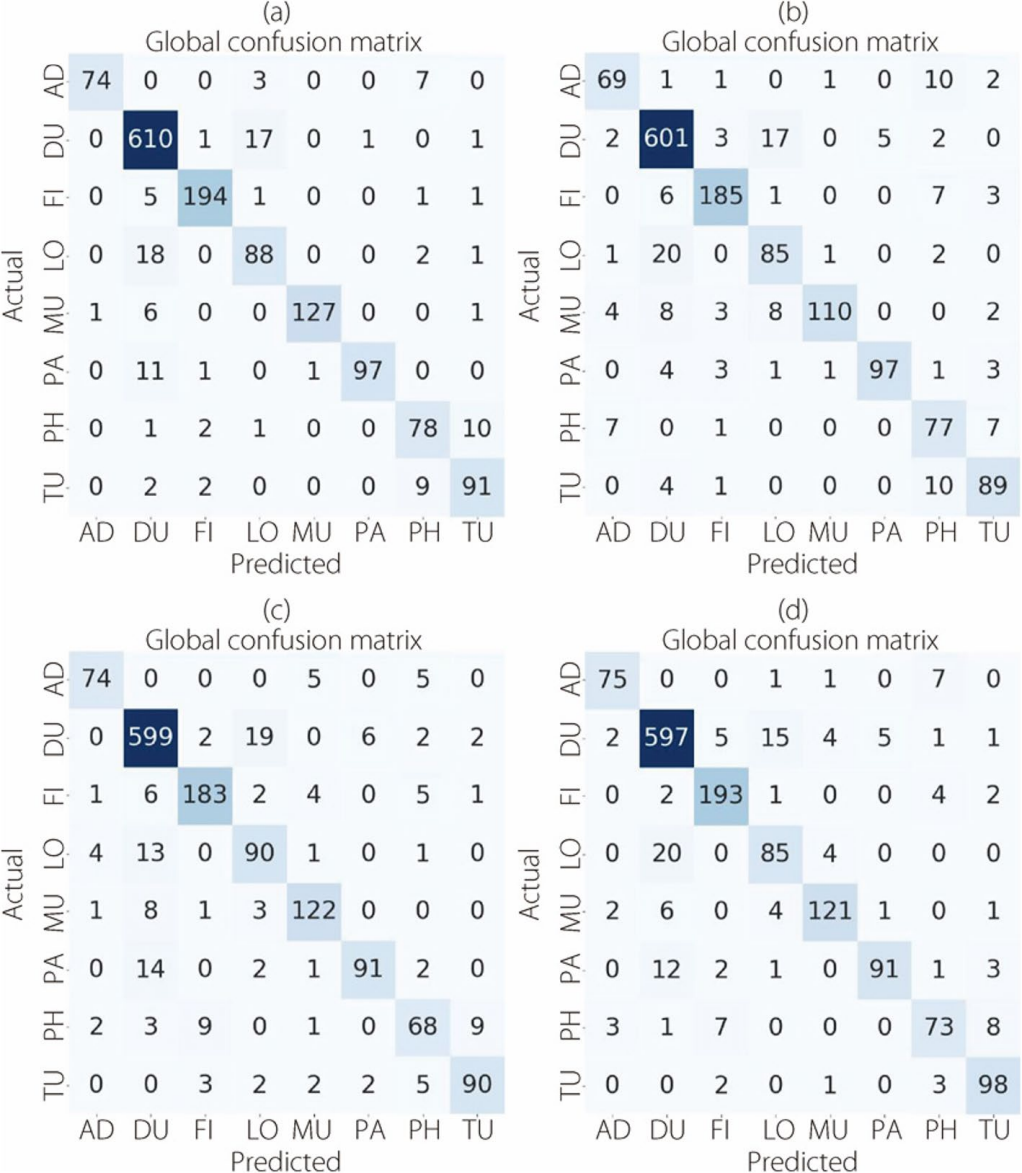
The multi-classification confusion matrices at 400× magnification for (**a**) ConvMixer, (**b**) TokenLearner, (**c**) ViT, and (**d**) TokenMixer models

Figure 15 shows confusion matrices for the classification of eight BC cancer types at a magnification level of 400×.

**Fig. 16:**
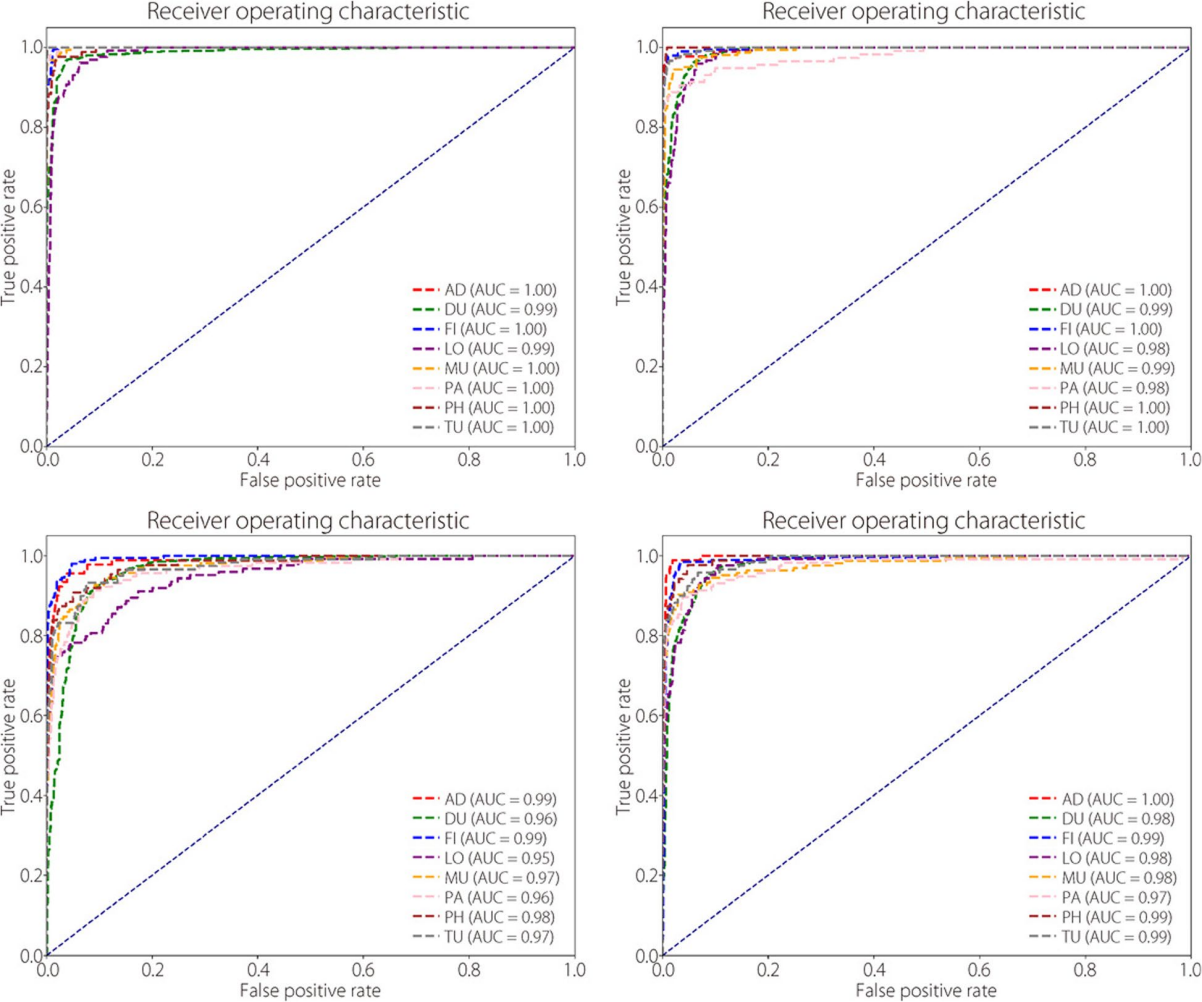
ROC curves of multi-classification for (**a**) ConvMixer, (**b**) TokenLearner, (**c**) ViT, and (**d**) TokenMixer at the best magnification

The ROC curves presented in Fig. 16 show the best performance of each model for the multi-classification.

## Discussion

**Fig. 17:**
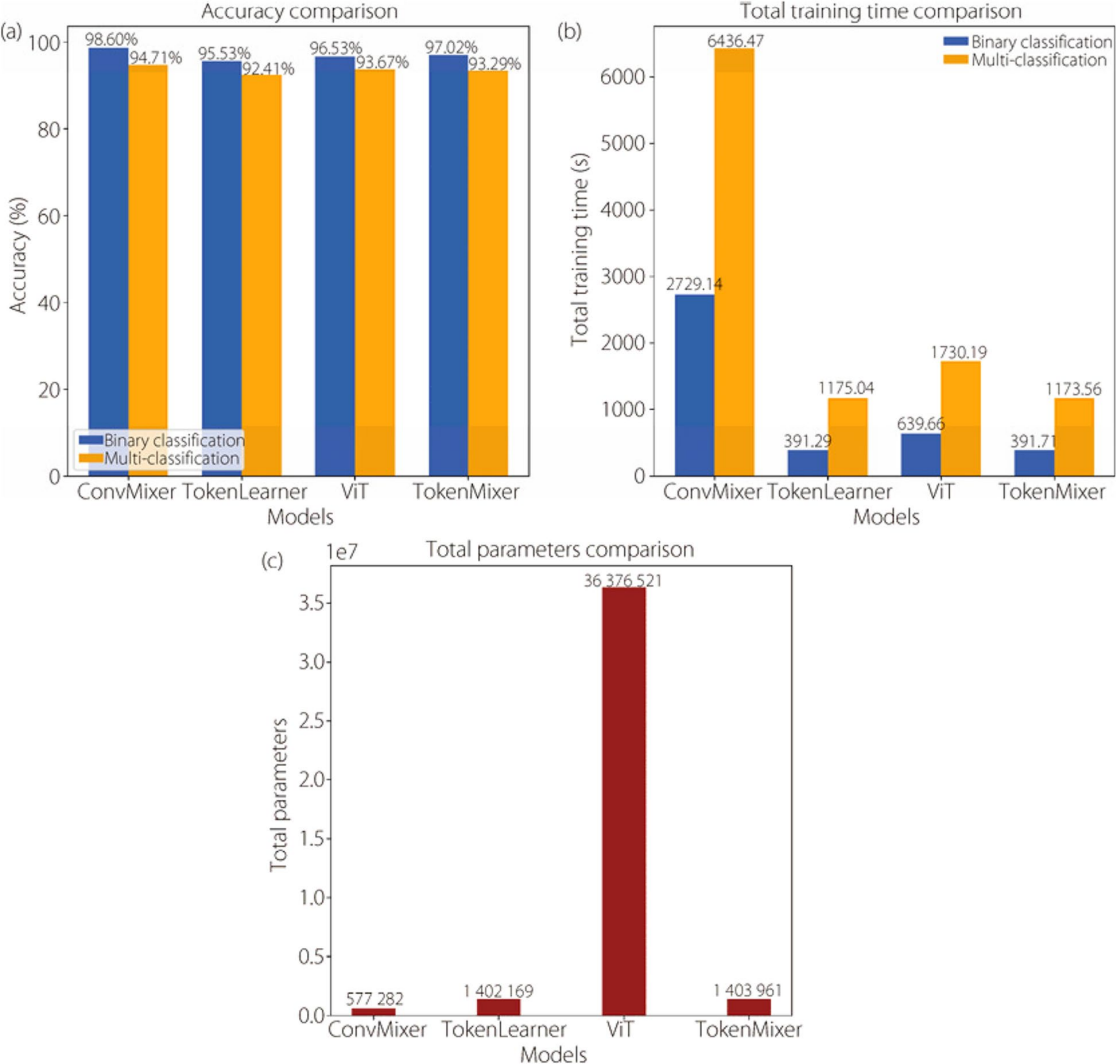
Summary results of the proposed methods

In this paper, we conducted experiments using four models to determine the most accurate magnification levels for binary and multi-classification of BC subtypes. To facilitate a better understanding and analysis, the experimental results are summarized in Fig. 17 that validates that TokenMixer was the superior classifier among all tested models. In binary classification, TokenMixer achieved an accuracy of 97.02% with a training time of 391.71 s, outperforming TokenLearner, which attained 95.53% accuracy in 391.29 s, and ViT, which achieved 96.53% accuracy in 639.66 s. However, it is worth noting that ViT involves a large number of parameters. ConvMixer achieved an accuracy of 98.60% but required a significantly longer training time of 2729.14 s, also exhibiting comparable multi-classification performance.

It is evident from Figs. 17a, b that ConvMixer, based on convolutional neural layers, outperformed the other models with a longer training time. However, ViT based on a self-attention mechanism also exhibited superior performance, but required a larger number of parameters, necessitating powerful computational resources for training.

The research question of whether it is possible to combine the strengths of CNNs and ViTs to construct hybrid and low-latency networks for medical data analysis is addressed. Experiments were conducted focusing on the TokenLearner model, which is based on tokenization mechanisms. The findings reveal significant promise in leveraging tokenization to streamline the ViT architecture and enhance its efficiency while maintaining accuracy. However, it is worth noting that the model requires further stabilization during training and improved classification accuracy. To address these gaps, the TokenMixer hybrid system is based on tokenization mechanisms along with attention maps generated by a depth-wise convolution 2D layer with input patches of size 14 × 14. With this method, the model may concentrate on certain areas or regions, which improves its capacity to extract significant features from tokens with inconsistent spatial information and provides stable training. Better performance with fewer parameters is achieved by using this tokenization strategy, which reduces computational complexity. This makes it the method of choice when combining convolutional with transformer layers. In the realm of BC histopathology images, ample opportunity remains to enhance the performance of Transformer-based models, especially in multi-classification scenarios that are crucial for the early detection of various cancer subtypes. This research suggests that developing lightweight Transformer models could lead to advancements beyond current standards. Prior research has mostly concentrated just of classification of benign and malignant classes. However, this study delved into multi-classification of BC using the BreakHis dataset, which includes eight classes (four benign and four malignant). In scenarios in which medical data are scarce, one approach that can be used is to employ a hierarchical framework for classification, consolidating benign classes into one category (one global benign and four malignant). It is important to explore whether the integration of hierarchical frameworks with tokenization Transformer techniques could significantly enhance their performance and generalization capabilities in classifying limited data. Compared to recent studies, the proposed approach shows significantly improved results, demonstrating the successful fusion of convolutional with transformer layers in identifying BC tumors. The markedly superior results of the proposed method underscore the effectiveness of TokenMixer in classifying breast tumors in histopathological images. Upon examining the findings vis-a-vis studies employing convolution ViT architectures [26, 34], it is evident that superior outcomes have been achieved (Table 11). This superiority can be attributed to several factors. First, the proposed method leverages the attention mechanism to generate spatial attention maps through convolution layers. These attention maps utilize tokenization to enhance feature extraction and minimize the input patches, thereby reducing the GPU resource complexity during training while maintaining high accuracy. Conversely, prior studies directly applied convolution layers to the input patches for feature extraction, resulting in longer training times and models with more parameters that are challenging to train without powerful GPUs. Second, in comparison to studies using convolution-based models [24, 25, 35–37, 41], the results of this study showcase comparable accuracy in BC classification in histopathological images. Notably, while some studies employed pretrained models, the proposed approach involves training the model from scratch, yet achieving equivalent accuracy performance.

**Table 11:** Comparative evaluation of earlier techniques for histopathological BC classification

Reference	Method	Classification	Performance	Magnification level
Ahmad et al. [24]	Efficient-Net	Binary	ACC 99.61%, precision 99.63%	40×
		Multi	ACC 96.99%, precision 97.47%	40×
Abimouloud et al. [25]	VIT	Binary	ACC 98.64% Time (H) = 0.14	400×
	CCT	Binary	ACC 96.99% Time (H) = 1.61	40×
	MVIT	Binary	ACC 97.52% Time (H) = 0.89	200×
	VIT	Multi	ACC 94.80%	400×
	CCT	Multi	ACC 84.60%	40×
	MVIT	Multi	ACC 87.84%	200×
Sriwastawa and Arul Jothi [26]	MaxViT	Binary	ACC 92.12% Time (H) = 8.83	
	ViT		ACC 86.86% Time (H) = 1.46	
	PiT		ACC 89.01% Time (H) = 8.49	
	CvT		ACC 91.41% Time (H) = 5.46	
	CrossFormer		ACC 90.39% Time (H) = 8.83	
	CrossViT		ACC 88.11% Time (H) = 1.46	
	NesT		ACC 91.66% Time (H) = 6.75	
	SepViT		ACC 87.45% Time (H) = 4.44	
Tummala et al. [34]	Swin transformer	Binary	ACC 99.60%	
		Multi	ACC 96.00%	40×
Boumaraf Said et al. [35]	ResNet 18	Binary	ACC 99.25%	40×
		Multi	ACC 94.49%	40×
Joseph et al. [36]	DNN	Binary	ACC 97.87%	40×
Srikantamurthy et al. [37]	CNN-LSTM	Binary	ACC 99.75%	100×
		Multi	ACC 96.30%	40×
Amin and Ahn [38]	FabNet	Multi	ACC 97.20%, precision 89.947%	400×
Hao et al. [39]	DenseNet201	Binary Binary	ACC 99.00%, precision 98.99% ACC 96.75%	40× 40×
Mahmud et al. [40]	AlexNet VGG16	Binary	ACC 91.37%	200×
Abunasser et al. [41]	BCCNN	Binary	ACC 98.30%	400×
	ConvMixer	Binary	ACC 98.60% Time (H) = 0.75	100×
	TokenLearner	Binary	ACC 95.53% Time (H) = 0.10	200×
	VIT	Binary	ACC 96.53% Time (H) = 0.17	200×
	TokenMixer	Binary	ACC 97.02% Time (H) = 0.10	200×
Our proposed systems	ConvMixer	Multi	ACC 94.71% Time (H) = 1.78	200×
	TokenLearner	Multi	ACC 92.41% Time (H) = 0.32	40×
	VIT	Multi	ACC 93.67% Time (H) = 0.48	40×
	TokenMixer	Multi	ACC 93.29% Time (H) = 0.32	40×

## Conclusions

As a first step toward cancer detection using histopathological images, a fast, accurate breast tumor classification approach has been proposed. The TokenMixer model based on a tokenization strategy and ViT attention techniques was presented. This model utilizes a two-step approach. The first step uses attention maps to extract features from input images and generate and tokenize input patches. The second stage involves training the ViT encoder with minimized input image patches extracted across the network. Notably, the TokenMixer model exhibited outstanding performance with reduced computational complexity, underscoring the strength of combining token ViTs infused with CNNs to construct powerful deep attention-convolution models for medical imaging. However, we have to admit that our method has certain inherent limitations.

Table 10 presents our examination of multi-classification performance, which indicates that all of the models we examined reached their maximum accuracy at a magnification of 40×. This finding is given by the challenges we faced with other magnification levels. Specifically, we grappled with a scarcity of samples and lower resolution images at these levels, which left us with a limited dataset for our multi-classification tasks.

This data limitation difficult loss impaired our models’ ability to effectively extract features from high-resolution images at various magnifications. Our results show a sobering review of the current state of both ViTs and CNNs when it comes to analyzing complex medical images, particularly in multi-classification scenarios with limited samples. This limitation appears to impair their ability to pick up on the visual features and subtle indicators of tumors that are important for accurately analyzing histopathological images. These findings emphasize the crucial role of data accessibility and quality in developing dependable medical image classification models. When utilizing limited or unbalanced datasets, models may face challenges in effective generalization, potentially resulting in overfitting and suboptimal performance. The following is a summary of the experiments conducted in this study:

- The ViT self-attention mechanism in ViTs does not provide substantial benefits compared to CNNs due to its higher complexity.
- The hybrid attention-convolution approach is a novel method that combines the strengths of both techniques in medical vision diagnosis.
- Tokenization mechanisms offer a promising solution for medical applications with restricted training data and limited computational GPU resources.

Future studies could explore the application of more algorithms in the clinical imaging domain to classify benign and malignant tumors and their subtypes. This could involve employing various models, such as vision language models, with datasets from different modalities, such as mammograms and MRI. Given the persistent challenge of data scarcity in medical imaging research, such investigations have significant potential to advance diagnostic capabilities and improve patient outcomes.

## Data Availability

The dataset analysed during the current study are available in: https://web.inf.ufpr.br/vri/databases/breast-cancer-histopathological-database-breakhis/.

## Abbreviations

ViT: Vision transformer
CNN: Convolutional neural network
BC: Breast cancer
SVM: Support vector machine
MRI: Magnetic resonance imaging
CCT: Compact convolutional transformer
MViT: Mobile vision transformer
CvT: Convolutional vision transformer
SepViT: Separable vision transformer
LSTM RNN: Long short-term memory recurrent neural network
GLCM: Gray-level co-occurrence matrix
AD: Adenocarcinoma
FI: Fibroadenoma
PH: Phyllodes tumor
TU: Tubular adenoma
DU: Ductal carcinoma
LO: Lobular carcinoma
MU: Mucinous carcinoma
PA: Papillary carcinoma
MLP: Multilayer perceptron
GELU: Gaussian Error Linear Unit
AUC: Area under the curve
MCC: Matthews correlation coefficient
TP: True positives
TN: True negatives
FP: False positives
FN: False negatives
